# Supplementary material for: How to not induce SNAs: The insufficiency of directional force
Source: PLoS One. 2023 Jun 29;18(6):e0288038. doi: 10.1371/journal.pone.0288038 (PMC10309995; doi:10.1371/journal.pone.0288038)
Supplement: S1 Table — shows the experimental factors of the SDA task. (DOCX) [file pone.0288038.s001.docx]

**S1 Table**

**S1 Table. Experimental Factors****.** S1 Table shows the experimental factors of the SDA task.

| Axes | Direction | Magnitude | Operator |
| --- | --- | --- | --- |
| Vertical | up | small/large | plus/minus |
|  | down | small/large | plus/minus |
| Horizontal | right | small/large | plus/minus |
|  | left | small/large | plus/minus |
