# Supplementary material for: How to not induce SNAs: The insufficiency of directional force
Source: PLoS One. 2023 Jun 29;18(6):e0288038. doi: 10.1371/journal.pone.0288038 (PMC10309995; doi:10.1371/journal.pone.0288038)
Supplement: S2 Table — shows the experimental stimuli of the SDA task. In total, 58 math problems (48 true and 10 distractors) were used as stimuli. The stimuli were presented twice across four blocks. Number magnitude was defined as small (numbers 1,2,3,4) and large (6,7,8,9). Number 5 was excluded from both operands and the result. (DOCX) [file pone.0288038.s002.docx]

**S2 Table**

**S2 Table.** **Experimental Stimuli of the SDA Experiment****.** S2 Table show the experimental stimuli of the SDA task. In total, 58 math problems (48 true and 10 distractors) were used as stimuli. The stimuli were presented twice across four blocks. Number magnitude was defined as small (numbers 1,2,3,4) and large (6,7,8,9). Number 5 was excluded from both operands and the result.

| True math problems | | | | | | | |
| --- | --- | --- | --- | --- | --- | --- | --- |
| *3+4=7* | *1+2=3* | *2-1=1* | *3+6=9* | *6-3=3* | *7+1=8* | *9-2=7* | *8-7=1* |
| *4+3=7* | *3+1=4* | *4-3=1* | *1+8=9* | *6-2=4* | *6+2=8* | *8-2=6* | *9-8=1* |
| *4+4=8* | *2+2=4* | *3-1=2* | *2+7=9* | *6-4=2* | *7+2=9* | *8-1=7* | *8-6=2* |
| *2+4=6* | *2+1=3* | *3-2=1* | *2+6=8* | *7-3=4* | *8+1=9* | *7-1=6* | *9-6=3* |
| *4+2=6* | *1+1=2* | *4-2=2* | *1+7=8* | *8-4=4* | *6+1=7* | *9-1=8* | *7-6=1* |
| *3+3=6* | *1+3=4* | *4-1=3* | *1+6=7* | *7-4=3* | *6+3=9* | *9-3=6* | *9-7=2* |
| Distractor math problems | | | |  |  |  |  |
| *4+2=8* | *3+1=2* | *4-3=3* | *1+7=6* | *9-3=4* | *6+2=6* | *7-3=6* | *9-7=4* |
|  |  | *2-1=3* |  |  |  | *8-4=6* |  |
