# Supplementary material for: How to not induce SNAs: The insufficiency of directional force
Source: PLoS One. 2023 Jun 29;18(6):e0288038. doi: 10.1371/journal.pone.0288038 (PMC10309995; doi:10.1371/journal.pone.0288038)
Supplement: S1 File — (DOCX) [file pone.0288038.s003.docx]

**S3 File. Data from individual Laboratories**

**Laboratory 1**

**Sample**

Laboratory 1 sample size is n = 54 (27 female, mean age 22.7, median 23, range 17-31). All participants were native German speakers and right-handed (lateral preference inventory showed a mean of 86.4 and a median of 88.9).

**Numbers Data RNG**

**Table 4. Absolut frequencies of randomly produced numbers in each direction.**

|  | *Direction* | | | | | |  |
| --- | --- | --- | --- | --- | --- | --- | --- |
| *Numbers* | ***Baseline*** | ***Left*** | ***Right*** | ***Down*** | ***Up*** | **Total** | |
| Large (6 - 9) | 2002 | 2004 | 1985 | 1993 | 1899 | 9883 | |
| Small (1 - 4) | 2124 | 2108 | 2164 | 2056 | 2035 | 10487 | |
| Total | 4126 | 4112 | 4149 | 4049 | 3934 | 20370 | |

**Force Data RNG**

**Table 2. Bayes factors of Bayesian t-tests of the RNG force data of a small vs. large number.** The analysis was performed with n = 51, 50, 48, and 49 for the directions left, right, up, and down, respectively. Cluster permutation analysis revealed relevant differences between small and large numbers during the time window 150-230 ms. However, Bayesian analysis reported only anecdotal evidence.

| Time windows  in ms | BF_01_ | | | |
| --- | --- | --- | --- | --- |
|  | Left | Right | Up | Down |
| 50-150 | 5.86 | 2.42 | 5.85 | 2.48 |
| 150-250 | 6.50 | 1.03 | 6.24 | 5.98 |
| 250-350 | 6.31 | 2.57 | 6.33 | 6.16 |
| 350-450 | 6.47 | 5.39 | 6.00 | 5.87 |
| 450-576 | 6.22 | 6.50 | 3.95 | 6.42 |
| 150-230 |  | 0.95 |  |  |

**Force Data SDA**

**Table 3. Bayes factors of Bayesian t-tests of the SDA force data for small vs. large operand 1.** The Operand analysis was performed with n = 43, 43, 51, and 47 for the directions left, right, up, and down, respectively.

| **Time windows in ms** | **B_01_ Operand 1** | | | |
| --- | --- | --- | --- | --- |
|  | Left | Right | Up | Down |
| 50 – 150 | 0.47 | 2.00 | 5.83 | 3.65 |
| 150 – 250 | 2.00 | 2.40 | 6.39 | 0.82 |
| 250 – 350 | 3.64 | 2.15 | 6.55 | 2.30 |
| 350 – 450 | 4.84 | 1.44 | 4.68 | 2.03 |
| 400 - 500 | 4.75 | 2.16 | 3.09 | 2.39 |

**Table 3. Bayes factors of Bayesian t-tests of the SDA force data for operator (plus vs. minus), operand 2 (small vs. large), and answer (small vs. large).** The operator sub table compares plus vs. minus after either a small or a large operand one. O2 and the result sub-tables report the results of small vs. large numbers within referred time windows after either a small or a large operand 1. The Operator analysis was performed with n = 42, 40, 48, and 46 for the directions left, right, up, and down, respectively. The O2 analysis was performed with n = 42, 38, 44, and 46 for the directions left, right, up, and down, respectively. The Answer analysis was performed with n = 41, 29, 41, and 44 for the directions left, right, up, and down, respectively. Cluster permutation analysis revealed relevant differences between plus and minus following a small operand 1 during the time window 344 - 417 ms. However, Bayesian analysis reported only anecdotal evidence.

|  | **BF01** | | | | | | | |
| --- | --- | --- | --- | --- | --- | --- | --- | --- |
| **Time windows in ms** |  |  |  |  |  |  |  |  |
|  | **Small O1 followed by:** | | | | **Large O1 followed by:** | | | |
|  | **Operator** | | | | **Operator** | | | |
|  | Left | Right | Up | Down | Left | Right | Up | Down |
| 50 – 150 | 5.94 | 0.47 | 3.24 | 6.25 | 5.87 | 5.59 | 1.17 | 2.02 |
| 150 – 250 | 5.93 | 0.93 | 3.43 | 6.25 | 5.60 | 2.59 | 1.24 | 1.74 |
| 250 – 350 | 4.44 | 0.42 | 3.18 | 6.24 | 5.98 | 2.32 | 0.78 | 2.46 |
| 350 – 450 | 4.32 | 0.18 | 3.38 | 6.14 | 5.98 | 1.71 | 0.45 | 1.69 |
| 400 - 500 | 4.99 | 0.27 | 3.84 | 6.03 | 5.98 | 2.36 | 1.14 | 1.20 |
| Perm. 344 - 417 |  | 0.86 |  |  |  |  |  |  |
|  | **O2** | | | | **O2** | | | |
|  | Left | Right | Up | Down | Left | Right | Up | Down |
| 50 – 150 | 5.80 | 5.43 | 5.47 | 6.03 | 5.83 | 5.26 | 4.19 | 6.04 |
| 150 – 250 | 5.87 | 5.59 | 5.46 | 6.22 | 5.78 | 4.88 | 2.89 | 6.25 |
| 250 – 350 | 5.97 | 2.75 | 5.81 | 6.07 | 5.96 | 5.36 | 2.89 | 6.23 |
| 350 – 450 | 5.75 | 2.85 | 5.70 | 6.01 | 5.69 | 5.22 | 5.75 | 5.99 |
| 400 - 500 | 5.80 | 3.10 | 5.60 | 6.20 | 5.63 | 5.36 | 5.41 | 5.91 |
|  |  |  |  |  |  |  |  |  |
|  | **Answer** | | | | **Answer** | | | |
|  | Left | Right | Up | Down | Left | Right | Up | Down |
| 50 – 150 | 1.35 | 4.52 | 5.93 | 5.88 | 3.64 | 4.45 | 0.77 | 1.08 |
| 150 – 250 | 1.02 | 4.33 | 5.79 | 5.81 | 3.27 | 2.00 | 1.10 | 1.52 |
| 250 – 350 | 1.13 | 5.04 | 5.93 | 6.10 | 4.15 | 2.89 | 1.19 | 3.14 |
| 350 – 450 | 0.44 | 4.93 | 5.78 | 6.11 | 3.96 | 1.71 | 1.75 | 3.40 |
| 400 - 500 | 0.36 | 4.87 | 5.85 | 6.10 | 5.00 | 2.23 | 1.98 | 2.73 |

**Laboratory 2**

Laboratory 2 sample size is n = 18 (14 female, mean age 23.9, median 23, range 18-35). All participants were native German speakers and right-handed (lateral preference inventory showed a mean of 70 and a median of 70).

**Numbers Data RNG**

**Table 4. Absolut frequencies of randomly produced numbers in each direction.**

|  | *Direction* | | | | | |  |
| --- | --- | --- | --- | --- | --- | --- | --- |
| *Numbers* | ***Baseline*** | ***Left*** | ***Right*** | ***Down*** | ***Up*** | **Total** | |
| Large (6 - 9) | 613 | 705 | 653 | 702 | 692 | 3365 | |
| Small (1 - 4) | 658 | 728 | 698 | 726 | 735 | 3545 | |
| Total | 1271 | 1433 | 1351 | 1428 | 1427 | 6910 | |

**Force Data RNG**

**Table 5. Results of Bayesian paired samples t-tests of the RNG force data for small vs. large numbers.** The analysis was performed with n = 18, 16, 15, and 18 for the directions left, right, up, and down, respectively.

| Time windows  in ms | BF_01_ | | | | | |
| --- | --- | --- | --- | --- | --- | --- |
|  | Left | Right | | Up | Down | |
| 50-150 ms | 4.11 | 3.86 | | 2.50 | 3.56 |  |
| 150-250 ms | 4.09 | 2.90 | | 3.13 | 2.50 |  |
| 250-350 ms | 3.78 | 2.66 | | 3.57 | 1.69 |  |
| 350-450 ms | 3.18 | 3.19 | | 3.46 | 2.42 |  |
| 450-486 ms | 3.89 | | 3.03 | 3.59 | 2.31 | |

**Force Data SDA**

**Table 6. Bayes factors of Bayesian t-tests of the SDA force data for a small vs. large operand 1.** The Operand 1 analysis was performed with n = 18, 17, 16, and 18 for the directions left, right, up, and down, respectively.

| **Time windows in ms** | **B_01_ Operand 1** | | | |
| --- | --- | --- | --- | --- |
|  | Left | Right | Up | Down |
| 50 – 150 | 3.88 | 4.01 | 3.91 | 4.11 |
| 150 – 250 | 4.00 | 4.01 | 3.82 | 3.79 |
| 250 – 350 | 3.82 | 3.94 | 3.19 | 3.27 |
| 350 – 450 | 3.79 | 3.99 | 3.25 | 2.86 |
| 400 - 500 | 4.10 | 3.98 | 3.36 | 3.00 |
|  |  |  |  |  |

**Table 6. Bayes factors of Bayesian t-tests of the SDA force data for operator (plus vs. minus), operand 2 (small vs. large), and answer (small vs. large).** The operator sub table compares plus vs. minus after either a small or a large operand 1. O2 and the result sub-tables report the results of small vs. large numbers within referred time windows after either a small or a large operand 1. The Operator analysis was performed with n = 18, 17, 14, and 17 for the directions left, right, up, and down, respectively. The O2 analysis was performed with n = 18, 14, 11, and 16 for the directions left, right, up, and down, respectively. The Answer analysis was performed with n = 18, 14, 5, and 16 for the directions left, right, up, and down, respectively.

|  | **BF01** | | | | | | | |
| --- | --- | --- | --- | --- | --- | --- | --- | --- |
| **Time windows in ms** |  |  |  |  |  |  |  |  |
|  | **Small O1 followed by:** | | | | **Large O1 followed by:** | | | |
|  | **Operator** | | | | **Operator** | | | |
|  | Left | Right | Up | Down | Left | Right | Up | Down |
| 50 – 150 | 2.51 | 3.86 | 3.67 | 3.36 | 2.75 | 4.00 | 3.52 | 1.15 |
| 150 – 250 | 4.08 | 3.65 | 3.19 | 3.38 | 2.86 | 4.01 | 3.54 | 2.13 |
| 250 – 350 | 4.11 | 3.22 | 3.60 | 3.46 | 2.76 | 3.82 | 2.16 | 3.24 |
| 350 – 450 | 4.11 | 2.64 | 3.65 | 3.98 | 4.06 | 3.63 | 1.92 | 3.60 |
| 400 - 500 | 3.99 | 2.81 | 3.49 | 4.01 | 4.11 | 3.71 | 1.97 | 3.93 |
|  |  |  |  |  |  |  |  |  |
|  | **O2** | | | | **O2** | | | |
| *N participants* | 18 | 14 | 11 | 16 | 18 | 14 | 11 | 16 |
|  | Left | Right | Up | Down | Left | Right | Up | Down |
| 50 – 150 | 3.62 | 2.19 | 3.28 | 3.58 | 3.07 | 2.91 | 3.34 | 1.37 |
| 150 – 250 | 4.00 | 2.78 | 3.17 | 3.28 | 2.65 | 2.64 | 3.01 | 2.15 |
| 250 – 350 | 4.06 | 2.94 | 3.15 | 3.92 | 2.96 | 3.13 | 2.58 | 3.00 |
| 350 – 450 | 4.07 | 3.54 | 2.47 | 3.82 | 3.71 | 3.37 | 2.37 | 2.98 |
| 400 - 500 | 4.07 | 3.54 | 2.27 | 3.86 | 3.91 | 3.04 | 2.53 | 2.83 |
|  |  |  |  |  |  |  |  |  |
|  | **Answer** | | | | **Answer** | | | |
| *N participants* | 18 | 14 | 5 | 16 | 41 | 14 | 5 | 16 |
|  | Left | Right | Up | Down | Left | Right | Up | Down |
| 50 – 150 | 0.51 | 3.36 | 1.64 | 2.96 | 3.93 | 0.60 | 0.03 | 3.64 |
| 150 – 250 | 0.38 | 3.47 | 1.34 | 3.78 | 4.08 | 0.52 | 0.29 | 3.24 |
| 250 – 350 | 0.62 | 3.20 | 1.12 | 3.92 | 4.07 | 0.40 | 0.76 | 2.86 |
| 350 – 450 | 0.35 | 3.62 | 1.32 | 3.72 | 3.88 | 0.36 | 0.58 | 2.47 |
| 400 - 500 | 0.35 | 3.67 | 1.46 | 3.68 | 4.11 | 0.49 | 0.41 | 2.85 |
