# Supplementary material for: How to not induce SNAs: The insufficiency of directional force
Source: PLoS One. 2023 Jun 29;18(6):e0288038. doi: 10.1371/journal.pone.0288038 (PMC10309995; doi:10.1371/journal.pone.0288038)
Supplement: S2 File — (DOCX) [file pone.0288038.s004.docx]

**S4 File**

**The Calibration Procedure**

A calibration procedure ensured that the initial force magnitude was standardized across all participants. During the training phase of calibration, participants pressed the sensor in the direction required by the current force direction condition. They were trained to maintain their force in the range between 1.5 N and 3 N. A circle on the screen provided feedback during calibration. When the force was within the limits of 1.5 N and 3 N, this circle turned green (otherwise it was grey when force was too little and red when force was too large). For this, participants had to continuously hold a steady force of 1.5 to 3N for 13 seconds. This procedure was repeated twice. Additionally, the calibration procedure was required to start each experimental test block (10 seconds). The rationale for choosing a baseline of 1.5 N was the so-called slip-ratio limit of about 27 g (27 mN) when holding an object of 68 g [1]; therefore, the force magnitude was never too low to allow dropping the sensor.

**References**

1. Turrell YN, Li FX, Wing AM. Estimating the minimum grip force required when grasping objects under impulsive loading conditions. Behav Res Methods, Instruments, Comput. 2001;33: 38–45. doi:10.3758/BF03195345
