# Supplementary material for: How to not induce SNAs: The insufficiency of directional force
Source: PLoS One. 2023 Jun 29;18(6):e0288038. doi: 10.1371/journal.pone.0288038 (PMC10309995; doi:10.1371/journal.pone.0288038)
Supplement: S3 File — (DOCX) [file pone.0288038.s005.docx]

**S5 File. Additional Information for the Analysis**

**Pre-processing of force data**

Pre-processing of force data was done based on the procedures described in Nazir et al. [1]. It was conducted in R (R Core Team, 2018) using home-made scripts. The pre-processing pipeline was similar for the SDA and RNG force data.

Only the Fz force vector was used for the analysis. First, each force signal component was low-pass filtered at 15 Hz with a fourth-order, zero-phase, low-pass Butterworth filter. Second, the force data was divided into epochs. Third, the data was baseline-corrected by subtracting the average of the force signal amplitude in the interval of the last 20 ms prior to the first stimulus (SDA) or the beep signal (RNG) onset. Fourth, an automatic artefact rejection was used to reduce force fluctuation variability. Signals outside the amplitude range of ±400 mN were rejected, in order to discard outliers relative to the initial force of 1.5 to 3 N. Originally, Nazir et al. [1] recommended to use ±200 mN, however, we adjusted the ranges to ±400 mN because we utilized longer trials that naturally have higher force fluctuation. Specifically, Nazir et al. [1] utilized 1 second trials while we had 2 seconds (RNG) and 2.5 seconds (SDA). Trials containing such signals were removed. Participants with artefact rejection rate of more than 20% were removed from the analysis. Finally, for SDA, the go-trials and false positive trials were removed from the analysis.

**Cluster Permutation Analysis**

The data aggregated over the respective conditions were submitted to cluster permutation analysis using the R package “permuco” [2] with stimulus value (small/large or respective combinations of operands, the operator and the answer) as a within-subject factor. We applied a total number of 5000 permutations and TFCE (Threshold-Free Cluster Enhancement) correction for multiple comparisons [following the procedure of 3,4]. In this study, it was used the permutation analysis as a diagnostic method for selection of specific time windows with significant differences between the conditions in both tasks. There were two significant time-windows resulting from the cluster permutation analysis from the laboratory 1 data. First, in the RNG task there was one significant time-window between 150 and 230 ms in the force directional condition to the right. However, it yielded anecdotal evidence of a BF01 = 0.95. Second, during the SDA task, also in the force directional condition to the right, there was a significant time-window between 344 and 417 ms after the operator onset. Again, the time-window yielded anecdotal evidence of a BF01 = 0.86.

**References**

1. Nazir TA, Hrycyk L, Moreau Q, Frak V, Cheylus A, Ott L, et al. A simple technique to study embodied language processes: the grip force sensor. Behav Res Methods. 2017;49: 61–73. doi:10.3758/s13428-015-0696-7

2. Frossard J, Renaud O. Permutation tests for regression, anova, and comparison of signals: The permuco package. J Stat Softw. 2021;99: 1–32. doi:10.18637/JSS.V099.I15

3. Miklashevsky A. Catch the star! Spatial information activates the manual motor system. Myachykov A, editor. PLoS One. 2022;17: e0262510. doi:10.1371/journal.pone.0262510

4. Miklashevsky A, Lindemann O, Fischer MH. The Force of Numbers: Investigating Manual Signatures of Embodied Number Processing. Front Hum Neurosci. 2021;14: 593. doi:10.3389/fnhum.2020.590508
