# Supplementary material for: How to not induce SNAs: The insufficiency of directional force
Source: PLoS One. 2023 Jun 29;18(6):e0288038. doi: 10.1371/journal.pone.0288038 (PMC10309995; doi:10.1371/journal.pone.0288038)
Supplement: S4 File — (DOCX) [file pone.0288038.s006.docx]

**S6 File**

**Pilot studies**

Two independent pilot studies with *n =* 7 (one laboratory; force direction conditions up and down) and *n =* 18 (mutual *n* from both laboratories; conditions left and right) were conducted. The data collected were used to ensure that the laboratory setup was working as intended in both locations. Bugs in the software were identified and eliminated. Adjustments in the experimental scripts were implemented to minimize the experimenter bias. Furthermore, participants’ subjective fatigue experiences were considered and self-paced breaks were implemented after feedback.
