# Supplementary material for: How to not induce SNAs: The insufficiency of directional force
Source: PLoS One. 2023 Jun 29;18(6):e0288038. doi: 10.1371/journal.pone.0288038 (PMC10309995; doi:10.1371/journal.pone.0288038)
Supplement: S5 File — (DOCX) [file pone.0288038.s007.docx]

**S7 File. Descriptive statistics of force data from individual Laboratories**

For all tables the means 1 to 5 are defined as 0-50, 50-150,150-250, 250-350, and 400-500 ms after the stimulus onset, respectively.

**Laboratory 1**

**RNG**

**Table 1. Descriptive statistics of the RNG force data of a small vs. large number for each direction.**

|  | **RNG Left** | | | |  |  | **RNG Right** | | | |  |  |
| --- | --- | --- | --- | --- | --- | --- | --- | --- | --- | --- | --- | --- |
|  |  |  |  |  | 95% Credible Interval | |  |  |  |  | 95% Credible Interval | |
|  | n | Mean | SD | SE | Lower | Upper | n | Mean | SD | SE | Lower | Upper |
| mean1_small | 51 | 5.77 | 7.63 | 1.07 | 3.62 | 7.91 | 50 | -3.83 | 9.56 | 1.35 | -6.54 | -1.11 |
| mean1_large | 51 | 5.37 | 7.47 | 1.05 | 3.27 | 7.48 | 50 | -2.00 | 10.63 | 1.50 | -5.02 | 1.02 |
| mean2_small | 51 | 9.19 | 14.19 | 1.99 | 5.20 | 13.19 | 50 | -4.89 | 16.34 | 2.31 | -9.54 | -0.25 |
| mean2_large | 51 | 9.34 | 15.66 | 2.19 | 4.94 | 13.75 | 50 | -1.66 | 17.64 | 2.50 | -6.67 | 3.36 |
| mean3_small | 51 | 11.45 | 23.77 | 3.33 | 4.76 | 18.13 | 50 | 7.45 | 26.34 | 3.73 | -0.04 | 14.94 |
| mean3_large | 51 | 11.88 | 24.68 | 3.46 | 4.94 | 18.82 | 50 | 10.90 | 26.96 | 3.81 | 3.24 | 18.56 |
| mean4_small | 51 | 10.59 | 32.11 | 4.50 | 1.56 | 19.63 | 50 | 17.08 | 35.96 | 5.09 | 6.86 | 27.30 |
| mean4_large | 51 | 10.91 | 33.08 | 4.63 | 1.61 | 20.21 | 50 | 18.93 | 35.80 | 5.06 | 8.76 | 29.11 |
| mean5_small | 51 | 6.74 | 39.92 | 5.59 | -4.48 | 17.97 | 50 | 28.36 | 43.11 | 6.10 | 16.11 | 40.62 |
| mean5_large | 51 | 7.46 | 41.70 | 5.84 | -4.27 | 19.18 | 50 | 28.37 | 42.74 | 6.05 | 16.22 | 40.52 |
|  | **RNG Down** | | | |  |  | **RNG Up** | | | |  |  |
|  |  |  |  |  | 95% Credible Interval | |  |  |  |  | 95% Credible Interval | |
|  | n | Mean | SD | SE | Lower | Upper | n | Mean | SD | SE | Lower | Upper |
| mean1_small | 49 | 5.09 | 7.46 | 1.07 | 2.95 | 7.23 | 48 | -3.03 | 12.78 | 1.84 | -6.74 | 0.68 |
| mean1_large | 49 | 6.36 | 7.46 | 1.07 | 4.21 | 8.50 | 48 | -3.59 | 11.86 | 1.71 | -7.03 | -0.14 |
| mean2_small | 49 | 10.55 | 11.11 | 1.59 | 7.36 | 13.74 | 48 | -4.86 | 23.25 | 3.36 | -11.61 | 1.90 |
| mean2_large | 49 | 11.05 | 11.88 | 1.70 | 7.64 | 14.46 | 48 | -5.29 | 22.69 | 3.28 | -11.88 | 1.30 |
| mean3_small | 49 | 16.77 | 12.98 | 1.85 | 13.04 | 20.50 | 48 | -0.55 | 32.42 | 4.68 | -9.96 | 8.87 |
| mean3_large | 49 | 16.36 | 15.76 | 2.25 | 11.84 | 20.89 | 48 | -0.27 | 33.20 | 4.79 | -9.91 | 9.38 |
| mean4_small | 49 | 19.12 | 19.19 | 2.74 | 13.61 | 24.63 | 48 | 3.80 | 33.17 | 4.79 | -5.84 | 13.43 |
| mean4_large | 49 | 18.51 | 22.38 | 3.20 | 12.09 | 24.94 | 48 | 4.56 | 35.88 | 5.18 | -5.86 | 14.97 |
| mean5_small | 49 | 18.37 | 26.89 | 3.84 | 10.64 | 26.09 | 48 | 10.36 | 34.43 | 4.97 | 0.37 | 20.36 |
| mean5_large | 49 | 18.50 | 30.75 | 4.39 | 9.67 | 27.33 | 48 | 8.24 | 35.91 | 5.18 | -2.19 | 18.67 |

**SDA**

**Table 2. Descriptive statistics of the SDA force data of a small vs. large operand 1 for each direction.**

|  | **O1 Left** | | | |  |  | **O1 Right** | | | |  |  |
| --- | --- | --- | --- | --- | --- | --- | --- | --- | --- | --- | --- | --- |
|  |  |  |  |  | 95% Credible Interval | |  |  |  |  | 95% Credible Interval | |
|  | n | Mean | SD | SE | Lower | Upper | n | Mean | SD | SE | Lower | Upper |
| mean1_small | 43 | 0.87 | 5.82 | 0.89 | -0.93 | 2.66 | 43 | -0.46 | 8.05 | 1.23 | -2.94 | 2.02 |
| mean1_large | 43 | -1.68 | 4.60 | 0.70 | -3.10 | -0.27 | 43 | -2.52 | 7.17 | 1.09 | -4.73 | -0.32 |
| mean2_small | 43 | 0.28 | 5.78 | 0.88 | -1.50 | 2.06 | 43 | -3.90 | 10.96 | 1.67 | -7.28 | -0.53 |
| mean2_large | 43 | -1.62 | 6.59 | 1.01 | -3.65 | 0.41 | 43 | -6.93 | 12.55 | 1.91 | -10.79 | -3.07 |
| mean3_small | 43 | 1.70 | 7.02 | 1.07 | -0.46 | 3.86 | 43 | -2.23 | 12.62 | 1.93 | -6.12 | 1.65 |
| mean3_large | 43 | -0.15 | 8.31 | 1.27 | -2.71 | 2.41 | 43 | -6.27 | 15.66 | 2.39 | -11.09 | -1.45 |
| mean4_small | 43 | 2.36 | 9.18 | 1.40 | -0.46 | 5.19 | 43 | 0.78 | 14.09 | 2.15 | -3.56 | 5.11 |
| mean4_large | 43 | 0.86 | 10.37 | 1.58 | -2.34 | 4.05 | 43 | -4.27 | 18.54 | 2.83 | -9.98 | 1.43 |
| mean5_small | 43 | 2.92 | 10.40 | 1.59 | -0.28 | 6.12 | 43 | 0.69 | 16.00 | 2.44 | -4.24 | 5.61 |
| mean5_large | 43 | 1.31 | 10.40 | 1.59 | -1.89 | 4.51 | 43 | -3.82 | 19.48 | 2.97 | -9.81 | 2.18 |
|  | **O1 Down** | | | |  |  | **O1 Up** | | | |  |  |
|  |  |  |  |  | 95% Credible Interval | |  |  |  |  | 95% Credible Interval | |
|  | n | Mean | SD | SE | Lower | Upper | n | Mean | SD | SE | Lower | Upper |
| mean1_small | 47 | -0.71 | 4.35 | 0.63 | -1.99 | 0.56 | 51 | -0.38 | 7.01 | 0.98 | -2.35 | 1.59 |
| mean1_large | 47 | 0.19 | 4.29 | 0.63 | -1.07 | 1.45 | 51 | -1.03 | 6.85 | 0.96 | -2.96 | 0.89 |
| mean2_small | 47 | -1.39 | 6.54 | 0.95 | -3.31 | 0.54 | 51 | -3.01 | 9.82 | 1.37 | -5.77 | -0.25 |
| mean2_large | 47 | 0.52 | 5.29 | 0.77 | -1.04 | 2.07 | 51 | -3.42 | 11.08 | 1.55 | -6.54 | -0.31 |
| mean3_small | 47 | 0.00 | 6.07 | 0.89 | -1.78 | 1.78 | 51 | -2.77 | 12.56 | 1.76 | -6.30 | 0.76 |
| mean3_large | 47 | 1.70 | 6.85 | 1.00 | -0.31 | 3.71 | 51 | -2.92 | 12.37 | 1.73 | -6.40 | 0.56 |
| mean4_small | 47 | 0.96 | 6.96 | 1.02 | -1.09 | 3.00 | 51 | -1.93 | 16.57 | 2.32 | -6.59 | 2.73 |
| mean4_large | 47 | 3.08 | 8.49 | 1.24 | 0.58 | 5.57 | 51 | 0.37 | 14.18 | 1.99 | -3.61 | 4.36 |
| mean5_small | 47 | 1.13 | 7.77 | 1.13 | -1.15 | 3.41 | 51 | -2.11 | 17.66 | 2.47 | -7.07 | 2.86 |
| mean5_large | 47 | 3.42 | 10.04 | 1.46 | 0.47 | 6.36 | 51 | 1.71 | 15.12 | 2.12 | -2.54 | 5.96 |

**Table 3. Descriptive statistics of the SDA force data of a plus vs. minus after a small operand 1 for each direction.**

|  | **Operator after small O1 Left** | | | |  |  | **Operator after small O1 Right** | | | |  |  |
| --- | --- | --- | --- | --- | --- | --- | --- | --- | --- | --- | --- | --- |
|  |  |  |  |  | 95% Credible Interval | |  |  |  |  | 95% Credible Interval | |
|  | n | Mean | SD | SE | Lower | Upper | n | Mean | SD | SE | Lower | Upper |
| mean1_minus | 48 | 2.19 | 29.84 | 4.31 | -6.48 | 10.85 | 40 | -12.08 | 30.13 | 4.76 | -21.72 | -2.45 |
| mean1_plus | 48 | -2.21 | 17.45 | 2.52 | -7.28 | 2.85 | 40 | 1.07 | 20.15 | 3.19 | -5.37 | 7.52 |
| mean2_minus | 48 | 2.15 | 33.02 | 4.77 | -7.43 | 11.74 | 40 | -14.31 | 32.96 | 5.21 | -24.86 | -3.77 |
| mean2_plus | 48 | -2.47 | 21.40 | 3.09 | -8.68 | 3.75 | 40 | -2.44 | 22.26 | 3.52 | -9.56 | 4.67 |
| mean3_minus | 48 | 1.10 | 38.37 | 5.54 | -10.04 | 12.24 | 40 | -20.27 | 37.54 | 5.94 | -32.27 | -8.26 |
| mean3_plus | 48 | -4.42 | 25.16 | 3.63 | -11.72 | 2.89 | 40 | -3.98 | 26.40 | 4.18 | -12.42 | 4.47 |
| mean4_minus | 48 | 1.52 | 42.77 | 6.17 | -10.90 | 13.94 | 40 | -20.18 | 39.55 | 6.25 | -32.83 | -7.53 |
| mean4_plus | 48 | -4.14 | 28.89 | 4.17 | -12.53 | 4.25 | 40 | -1.19 | 29.11 | 4.60 | -10.50 | 8.12 |
| mean5_minus | 48 | 1.68 | 44.23 | 6.38 | -11.17 | 14.52 | 40 | -19.73 | 40.56 | 6.41 | -32.70 | -6.76 |
| mean5_plus | 48 | -3.50 | 30.16 | 4.35 | -12.25 | 5.26 | 40 | -0.94 | 30.95 | 4.89 | -10.84 | 8.95 |
|  | **Operator after small O1 Down** | | | |  |  | **Operator after small O1 Up** | | | |  |  |
|  |  |  |  |  | 95% Credible Interval | |  |  |  |  | 95% Credible Interval | |
|  | n | Mean | SD | SE | Lower | Upper | n | Mean | SD | SE | Lower | Upper |
| mean1_minus | 46 | 0.77 | 14.95 | 2.20 | -3.67 | 5.21 | 48 | 2.19 | 29.84 | 4.31 | -6.48 | 10.85 |
| mean1_plus | 46 | 0.83 | 12.02 | 1.77 | -2.74 | 4.40 | 48 | -2.21 | 17.45 | 2.52 | -7.28 | 2.85 |
| mean2_minus | 46 | 0.89 | 15.50 | 2.29 | -3.72 | 5.49 | 48 | 2.15 | 33.02 | 4.77 | -7.43 | 11.74 |
| mean2_plus | 46 | 0.96 | 12.09 | 1.78 | -2.63 | 4.55 | 48 | -2.47 | 21.40 | 3.09 | -8.68 | 3.75 |
| mean3_minus | 46 | 0.67 | 17.39 | 2.56 | -4.49 | 5.84 | 48 | 1.10 | 38.37 | 5.54 | -10.04 | 12.24 |
| mean3_plus | 46 | 0.81 | 12.04 | 1.78 | -2.76 | 4.39 | 48 | -4.42 | 25.16 | 3.63 | -11.72 | 2.89 |
| mean4_minus | 46 | 0.86 | 20.00 | 2.95 | -5.08 | 6.80 | 48 | 1.52 | 42.77 | 6.17 | -10.90 | 13.94 |
| mean4_plus | 46 | 1.42 | 12.68 | 1.87 | -2.35 | 5.18 | 48 | -4.14 | 28.89 | 4.17 | -12.53 | 4.25 |
| mean5_minus | 46 | 0.77 | 20.50 | 3.02 | -5.32 | 6.86 | 48 | 1.68 | 44.23 | 6.38 | -11.17 | 14.52 |
| mean5_plus | 46 | 1.60 | 13.48 | 1.99 | -2.40 | 5.61 | 48 | -3.50 | 30.16 | 4.35 | -12.25 | 5.26 |

**Table 4. Descriptive statistics of the SDA force data of a plus vs. minus after a large operand 1 for each direction.**

|  | **Operator after large O1 Left** | | | |  |  | **Operator after large O1 Right** | | | |  |  |
| --- | --- | --- | --- | --- | --- | --- | --- | --- | --- | --- | --- | --- |
|  |  |  |  |  | 95% Credible Interval | |  |  |  |  | 95% Credible Interval | |
|  | n | Mean | SD | SE | Lower | Upper | n | Mean | SD | SE | Lower | Upper |
| mean1_minus | 48 | -0.1 | 19.3 | 2.8 | -5.7 | 5.5 | 40 | -5.66 | 20.41 | 3.23 | -12.19 | 0.87 |
| mean1_plus | 48 | 5.5 | 23.0 | 3.3 | -1.1 | 12.2 | 40 | -4.04 | 32.78 | 5.18 | -14.52 | 6.44 |
| mean2_minus | 48 | -2.0 | 21.9 | 3.2 | -8.3 | 4.4 | 40 | -8.74 | 23.99 | 3.79 | -16.41 | -1.06 |
| mean2_plus | 48 | 4.6 | 26.1 | 3.8 | -2.9 | 12.2 | 40 | -1.12 | 34.90 | 5.52 | -12.28 | 10.04 |
| mean3_minus | 48 | -3.4 | 26.0 | 3.8 | -10.9 | 4.2 | 40 | -9.66 | 22.67 | 3.58 | -16.91 | -2.41 |
| mean3_plus | 48 | 5.3 | 31.7 | 4.6 | -3.9 | 14.5 | 40 | -1.66 | 35.81 | 5.66 | -13.11 | 9.79 |
| mean4_minus | 48 | -2.4 | 30.5 | 4.4 | -11.3 | 6.4 | 40 | -10.07 | 22.65 | 3.58 | -17.31 | -2.82 |
| mean4_plus | 48 | 8.1 | 36.0 | 5.2 | -2.4 | 18.5 | 40 | -0.26 | 39.43 | 6.23 | -12.87 | 12.35 |
| mean5_minus | 48 | -1.2 | 31.8 | 4.6 | -10.5 | 8.0 | 40 | -10.04 | 24.24 | 3.83 | -17.79 | -2.29 |
| mean5_plus | 48 | 7.5 | 37.1 | 5.3 | -3.2 | 18.3 | 40 | -1.63 | 39.64 | 6.27 | -14.31 | 11.05 |
|  | **Operator after large O1 Down** | | | |  |  | **Operator after large O1 Up** | | | |  |  |
|  |  |  |  |  | 95% Credible Interval | |  |  |  |  | 95% Credible Interval | |
|  | n | Mean | SD | SE | Lower | Upper | n | Mean | SD | SE | Lower | Upper |
| mean1_minus | 46 | 2.78 | 11.83 | 1.74 | -0.73 | 6.29 | 48 | -0.14 | 19.32 | 2.79 | -5.75 | 5.47 |
| mean1_plus | 46 | -1.43 | 14.79 | 2.18 | -5.82 | 2.96 | 48 | 5.55 | 23.04 | 3.33 | -1.14 | 12.24 |
| mean2_minus | 46 | 2.78 | 14.14 | 2.09 | -1.42 | 6.98 | 48 | -1.97 | 21.95 | 3.17 | -8.35 | 4.40 |
| mean2_plus | 46 | -2.52 | 15.90 | 2.35 | -7.24 | 2.20 | 48 | 4.62 | 26.06 | 3.76 | -2.95 | 12.19 |
| mean3_minus | 46 | 2.41 | 16.41 | 2.42 | -2.46 | 7.28 | 48 | -3.39 | 26.02 | 3.76 | -10.95 | 4.16 |
| mean3_plus | 46 | -2.38 | 16.47 | 2.43 | -7.27 | 2.51 | 48 | 5.30 | 31.72 | 4.58 | -3.91 | 14.51 |
| mean4_minus | 46 | 2.63 | 18.02 | 2.66 | -2.72 | 7.98 | 48 | -2.44 | 30.49 | 4.40 | -11.29 | 6.42 |
| mean4_plus | 46 | -2.73 | 17.02 | 2.51 | -7.79 | 2.33 | 48 | 8.07 | 35.98 | 5.19 | -2.38 | 18.52 |
| mean5_minus | 46 | 2.52 | 18.56 | 2.74 | -3.00 | 8.03 | 48 | -1.22 | 31.84 | 4.60 | -10.46 | 8.03 |
| mean5_plus | 46 | -3.16 | 18.23 | 2.69 | -8.57 | 2.25 | 48 | 7.52 | 37.06 | 5.35 | -3.25 | 18.28 |

**Table 5. Descriptive statistics of the SDA force data of a small vs. large operand 2 after a small operand 1 for each direction.**

|  | **O2 after small O1 Left** | | | |  |  | **O2 after small O1 Right** | | | |  |  |
| --- | --- | --- | --- | --- | --- | --- | --- | --- | --- | --- | --- | --- |
|  |  |  |  |  | 95% Credible Interval | |  |  |  |  | 95% Credible Interval | |
|  | n | Mean | SD | SE | Lower | Upper | n | Mean | SD | SE | Lower | Upper |
| mean1_small | 44 | -1.6 | 25.3 | 3.8 | -9.3 | 6.1 | 38 | -6.49 | 37.85 | 6.14 | -18.93 | 5.96 |
| mean1_large | 44 | 1.4 | 36.7 | 5.5 | -9.8 | 12.6 | 38 | -4.48 | 33.42 | 5.42 | -15.46 | 6.50 |
| mean2_small | 44 | -1.5 | 25.6 | 3.9 | -9.3 | 6.3 | 38 | -6.73 | 38.79 | 6.29 | -19.48 | 6.02 |
| mean2_large | 44 | 1.6 | 39.0 | 5.9 | -10.3 | 13.4 | 38 | -8.07 | 33.85 | 5.49 | -19.19 | 3.05 |
| mean3_small | 44 | -2.0 | 27.4 | 4.1 | -10.3 | 6.3 | 38 | -4.58 | 39.83 | 6.46 | -17.67 | 8.51 |
| mean3_large | 44 | 0.1 | 39.6 | 6.0 | -12.0 | 12.1 | 38 | -12.64 | 38.62 | 6.27 | -25.34 | 0.05 |
| mean4_small | 44 | -0.7 | 27.4 | 4.1 | -9.0 | 7.6 | 38 | 0.23 | 45.45 | 7.37 | -14.71 | 15.16 |
| mean4_large | 44 | 1.6 | 40.3 | 6.1 | -10.6 | 13.9 | 38 | -7.95 | 36.80 | 5.97 | -20.04 | 4.15 |
| mean5_small | 44 | 0.3 | 28.0 | 4.2 | -8.3 | 8.8 | 38 | 1.24 | 47.28 | 7.67 | -14.30 | 16.78 |
| mean5_large | 44 | 2.9 | 42.0 | 6.3 | -9.9 | 15.7 | 38 | -6.87 | 39.74 | 6.45 | -19.94 | 6.19 |
|  | **O2 after small O1 Down** | | | |  |  | **O2 after small O1 Up** | | | |  |  |
|  |  |  |  |  | 95% Credible Interval | |  |  |  |  | 95% Credible Interval | |
|  | n | Mean | SD | SE | Lower | Upper | n | Mean | SD | SE | Lower | Upper |
| mean1_small | 46 | 0.70 | 18.65 | 2.75 | -4.84 | 6.23 | 44 | -1.61 | 25.33 | 3.82 | -9.31 | 6.09 |
| mean1_large | 46 | -0.24 | 21.26 | 3.13 | -6.55 | 6.07 | 44 | 1.40 | 36.74 | 5.54 | -9.77 | 12.57 |
| mean2_small | 46 | -0.52 | 20.40 | 3.01 | -6.58 | 5.53 | 44 | -1.48 | 25.62 | 3.86 | -9.27 | 6.31 |
| mean2_large | 46 | -0.88 | 23.13 | 3.41 | -7.75 | 5.99 | 44 | 1.57 | 39.04 | 5.89 | -10.30 | 13.44 |
| mean3_small | 46 | -1.81 | 21.73 | 3.20 | -8.26 | 4.64 | 44 | -1.97 | 27.35 | 4.12 | -10.29 | 6.34 |
| mean3_large | 46 | -0.91 | 24.08 | 3.55 | -8.06 | 6.24 | 44 | 0.06 | 39.58 | 5.97 | -11.97 | 12.09 |
| mean4_small | 46 | -2.31 | 23.42 | 3.45 | -9.26 | 4.65 | 44 | -0.71 | 27.44 | 4.14 | -9.05 | 7.64 |
| mean4_large | 46 | -1.22 | 25.18 | 3.71 | -8.69 | 6.26 | 44 | 1.61 | 40.28 | 6.07 | -10.64 | 13.85 |
| mean5_small | 46 | -2.20 | 24.21 | 3.57 | -9.39 | 4.99 | 44 | 0.27 | 28.04 | 4.23 | -8.25 | 8.80 |
| mean5_large | 46 | -1.67 | 25.77 | 3.80 | -9.32 | 5.98 | 44 | 2.92 | 42.02 | 6.34 | -9.86 | 15.69 |

**Table 6. Descriptive statistics of the SDA force data of a small vs. large operand 2 after a large operand 1 for each direction.**

|  | **O2 after large O1 Left** | | | |  |  | **O2 after large O1 Right** | | | |  |  |
| --- | --- | --- | --- | --- | --- | --- | --- | --- | --- | --- | --- | --- |
|  |  |  |  |  | 95% Credible Interval | |  |  |  |  | 95% Credible Interval | |
|  | n | Mean | SD | SE | Lower | Upper | n | Mean | SD | SE | Lower | Upper |
| mean1_small | 44 | -1.2 | 32.2 | 4.9 | -11.0 | 8.6 | 38 | -11.88 | 27.86 | 4.52 | -21.04 | -2.72 |
| mean1_large | 44 | 3.0 | 31.2 | 4.7 | -6.5 | 12.5 | 38 | -14.57 | 39.95 | 6.48 | -27.70 | -1.44 |
| mean2_small | 44 | -3.7 | 34.0 | 5.1 | -14.1 | 6.6 | 38 | -13.50 | 29.20 | 4.74 | -23.10 | -3.90 |
| mean2_large | 44 | 2.3 | 32.1 | 4.8 | -7.5 | 12.1 | 38 | -16.71 | 36.12 | 5.86 | -28.58 | -4.84 |
| mean3_small | 44 | -4.5 | 35.1 | 5.3 | -15.2 | 6.2 | 38 | -14.59 | 30.14 | 4.89 | -24.49 | -4.68 |
| mean3_large | 44 | 2.2 | 34.6 | 5.2 | -8.3 | 12.7 | 38 | -16.53 | 37.31 | 6.05 | -28.79 | -4.27 |
| mean4_small | 44 | -0.3 | 36.0 | 5.4 | -11.2 | 10.7 | 38 | -11.13 | 31.69 | 5.14 | -21.55 | -0.72 |
| mean4_large | 44 | 1.8 | 37.5 | 5.6 | -9.6 | 13.2 | 38 | -13.83 | 40.00 | 6.49 | -26.97 | -0.68 |
| mean5_small | 44 | 1.1 | 37.5 | 5.7 | -10.3 | 12.5 | 38 | -9.87 | 32.34 | 5.25 | -20.50 | 0.76 |
| mean5_large | 44 | 4.2 | 39.7 | 6.0 | -7.8 | 16.3 | 38 | -12.22 | 39.86 | 6.47 | -25.32 | 0.88 |
|  | **O2 after large O1 Down** | | | |  |  | **O2 after large O1 Up** | | | |  |  |
|  |  |  |  |  | 95% Credible Interval | |  |  |  |  | 95% Credible Interval | |
|  | n | Mean | SD | SE | Lower | Upper | n | Mean | SD | SE | Lower | Upper |
| mean1_small | 46 | 2.24 | 18.01 | 2.66 | -3.11 | 7.59 | 44 | -1.23 | 32.23 | 4.86 | -11.02 | 8.57 |
| mean1_large | 46 | 3.16 | 28.40 | 4.19 | -5.27 | 11.60 | 44 | 2.98 | 31.16 | 4.70 | -6.49 | 12.46 |
| mean2_small | 46 | 1.47 | 18.98 | 2.80 | -4.16 | 7.11 | 44 | -3.74 | 33.98 | 5.12 | -14.07 | 6.60 |
| mean2_large | 46 | 1.37 | 29.00 | 4.28 | -7.24 | 9.99 | 44 | 2.32 | 32.13 | 4.84 | -7.45 | 12.08 |
| mean3_small | 46 | 0.68 | 19.75 | 2.91 | -5.19 | 6.54 | 44 | -4.48 | 35.12 | 5.30 | -15.16 | 6.19 |
| mean3_large | 46 | 0.38 | 29.07 | 4.29 | -8.26 | 9.01 | 44 | 2.19 | 34.60 | 5.22 | -8.33 | 12.71 |
| mean4_small | 46 | 1.62 | 20.89 | 3.08 | -4.58 | 7.83 | 44 | -0.25 | 36.00 | 5.43 | -11.20 | 10.70 |
| mean4_large | 46 | 0.54 | 29.56 | 4.36 | -8.24 | 9.32 | 44 | 1.82 | 37.46 | 5.65 | -9.57 | 13.21 |
| mean5_small | 46 | 1.93 | 21.26 | 3.14 | -4.38 | 8.25 | 44 | 1.12 | 37.49 | 5.65 | -10.28 | 12.51 |
| mean5_large | 46 | 0.72 | 29.68 | 4.38 | -8.10 | 9.53 | 44 | 4.23 | 39.67 | 5.98 | -7.83 | 16.29 |

**Table 7. Descriptive statistics of the SDA force data of a small vs. large answer after a small operand 1 for each direction.**

|  | **Answer after small O1 Left** | | | |  |  | **Answer after small O1 Right** | | | |  |  |
| --- | --- | --- | --- | --- | --- | --- | --- | --- | --- | --- | --- | --- |
|  |  |  |  |  | 95% Credible Interval | |  |  |  |  | 95% Credible Interval | |
|  | n | Mean | SD | SE | Lower | Upper | n | Mean | SD | SE | Lower | Upper |
| mean1_small | 41 | 2.38 | 46.18 | 7.21 | -12.20 | 16.96 | 29 | -3.76 | 36.35 | 6.75 | -17.59 | 10.06 |
| mean1_large | 41 | 2.49 | 41.25 | 6.44 | -10.53 | 15.51 | 29 | 0.61 | 53.10 | 9.86 | -19.59 | 20.81 |
| mean2_small | 41 | 0.48 | 47.33 | 7.39 | -14.46 | 15.42 | 29 | -11.05 | 38.25 | 7.10 | -25.60 | 3.50 |
| mean2_large | 41 | -1.37 | 44.26 | 6.91 | -15.34 | 12.60 | 29 | -6.17 | 55.42 | 10.29 | -27.25 | 14.91 |
| mean3_small | 41 | -6.71 | 48.04 | 7.50 | -21.87 | 8.46 | 29 | -18.39 | 37.36 | 6.94 | -32.60 | -4.18 |
| mean3_large | 41 | -6.46 | 44.41 | 6.94 | -20.47 | 7.56 | 29 | -17.60 | 55.33 | 10.28 | -38.65 | 3.45 |
| mean4_small | 41 | -8.66 | 49.76 | 7.77 | -24.37 | 7.05 | 29 | -16.53 | 36.04 | 6.69 | -30.24 | -2.82 |
| mean4_large | 41 | -6.60 | 44.15 | 6.89 | -20.54 | 7.33 | 29 | -18.65 | 53.97 | 10.02 | -39.18 | 1.88 |
| mean5_small | 41 | -9.60 | 49.74 | 7.77 | -25.30 | 6.10 | 29 | -14.89 | 35.19 | 6.54 | -28.28 | -1.51 |
| mean5_large | 41 | -8.13 | 44.17 | 6.90 | -22.07 | 5.82 | 29 | -17.49 | 52.71 | 9.79 | -37.54 | 2.56 |
|  | **Answer after small O1 Down** | | | |  |  | **Answer after small O1 Up** | | | |  |  |
|  |  |  |  |  | 95% Credible Interval | |  |  |  |  | 95% Credible Interval | |
|  | n | Mean | SD | SE | Lower | Upper | n | Mean | SD | SE | Lower | Upper |
| mean1_small | 44 | 0.35 | 29.00 | 4.37 | -8.47 | 9.17 | 41 | 2.38 | 46.18 | 7.21 | -12.20 | 16.96 |
| mean1_large | 44 | -1.40 | 33.86 | 5.11 | -11.70 | 8.89 | 41 | 2.49 | 41.25 | 6.44 | -10.53 | 15.51 |
| mean2_small | 44 | 0.09 | 29.64 | 4.47 | -8.92 | 9.10 | 41 | 0.48 | 47.33 | 7.39 | -14.46 | 15.42 |
| mean2_large | 44 | -1.85 | 33.96 | 5.12 | -12.17 | 8.48 | 41 | -1.37 | 44.26 | 6.91 | -15.34 | 12.60 |
| mean3_small | 44 | -1.34 | 29.82 | 4.50 | -10.41 | 7.73 | 41 | -6.71 | 48.04 | 7.50 | -21.87 | 8.46 |
| mean3_large | 44 | -1.79 | 32.92 | 4.96 | -11.80 | 8.22 | 41 | -6.46 | 44.41 | 6.94 | -20.47 | 7.56 |
| mean4_small | 44 | -2.28 | 30.13 | 4.54 | -11.44 | 6.88 | 41 | -8.66 | 49.76 | 7.77 | -24.37 | 7.05 |
| mean4_large | 44 | -1.96 | 32.23 | 4.86 | -11.76 | 7.84 | 41 | -6.60 | 44.15 | 6.89 | -20.54 | 7.33 |
| mean5_small | 44 | -3.42 | 30.52 | 4.60 | -12.70 | 5.86 | 41 | -9.60 | 49.74 | 7.77 | -25.30 | 6.10 |
| mean5_large | 44 | -3.85 | 32.48 | 4.90 | -13.72 | 6.03 | 41 | -8.13 | 44.17 | 6.90 | -22.07 | 5.82 |

**Table 8. Descriptive statistics of the SDA force data of a small vs. large answer after a large operand 1 for each direction.**

|  | **Answer after large O1 Left** | | | |  |  | **Answer after large O1 Right** | | | |  |  |
| --- | --- | --- | --- | --- | --- | --- | --- | --- | --- | --- | --- | --- |
|  |  |  |  |  | 95% Credible Interval | |  |  |  |  | 95% Credible Interval | |
|  | n | Mean | SD | SE | Lower | Upper | n | Mean | SD | SE | Lower | Upper |
| mean1_small | 41 | 0.86 | 35.68 | 5.57 | -10.41 | 12.12 | 29 | -6.18 | 29.96 | 5.56 | -17.58 | 5.21 |
| mean1_large | 41 | 19.24 | 52.41 | 8.19 | 2.70 | 35.78 | 29 | -2.71 | 41.38 | 7.68 | -18.45 | 13.03 |
| mean2_small | 41 | -0.78 | 36.00 | 5.62 | -12.14 | 10.59 | 29 | -15.87 | 30.37 | 5.64 | -27.42 | -4.32 |
| mean2_large | 41 | 15.08 | 51.70 | 8.07 | -1.24 | 31.40 | 29 | -5.35 | 44.18 | 8.20 | -22.16 | 11.45 |
| mean3_small | 41 | -5.34 | 35.50 | 5.54 | -16.55 | 5.86 | 29 | -20.57 | 30.96 | 5.75 | -32.35 | -8.79 |
| mean3_large | 41 | 9.94 | 52.34 | 8.17 | -6.58 | 26.46 | 29 | -13.44 | 43.00 | 7.98 | -29.80 | 2.91 |
| mean4_small | 41 | -5.79 | 37.17 | 5.81 | -17.52 | 5.94 | 29 | -18.68 | 28.26 | 5.25 | -29.43 | -7.93 |
| mean4_large | 41 | 7.60 | 53.61 | 8.37 | -9.32 | 24.52 | 29 | -10.59 | 38.78 | 7.20 | -25.34 | 4.17 |
| mean5_small | 41 | -6.18 | 38.54 | 6.02 | -18.34 | 5.99 | 29 | -16.01 | 27.27 | 5.07 | -26.39 | -5.64 |
| mean5_large | 41 | 6.81 | 53.45 | 8.35 | -10.06 | 23.68 | 29 | -8.79 | 38.19 | 7.09 | -23.32 | 5.74 |
|  | **Answer after large O1 Down** | | | |  |  | **Answer after large O1 Up** | | | |  |  |
|  |  |  |  |  | 95% Credible Interval | |  |  |  |  | 95% Credible Interval | |
|  | n | Mean | SD | SE | Lower | Upper | n | Mean | SD | SE | Lower | Upper |
| mean1_small | 44 | -2.12 | 32.67 | 4.93 | -12.05 | 7.81 | 41 | 0.86 | 35.68 | 5.57 | -10.41 | 12.12 |
| mean1_large | 44 | 8.84 | 26.41 | 3.98 | 0.81 | 16.87 | 41 | 19.24 | 52.41 | 8.19 | 2.70 | 35.78 |
| mean2_small | 44 | -1.64 | 32.48 | 4.90 | -11.52 | 8.24 | 41 | -0.78 | 36.00 | 5.62 | -12.14 | 10.59 |
| mean2_large | 44 | 7.70 | 25.89 | 3.90 | -0.17 | 15.57 | 41 | 15.08 | 51.70 | 8.07 | -1.24 | 31.40 |
| mean3_small | 44 | -1.59 | 31.12 | 4.69 | -11.05 | 7.87 | 41 | -5.34 | 35.50 | 5.54 | -16.55 | 5.86 |
| mean3_large | 44 | 4.57 | 25.10 | 3.78 | -3.06 | 12.21 | 41 | 9.94 | 52.34 | 8.17 | -6.58 | 26.46 |
| mean4_small | 44 | -2.80 | 29.70 | 4.48 | -11.83 | 6.23 | 41 | -5.79 | 37.17 | 5.81 | -17.52 | 5.94 |
| mean4_large | 44 | 2.84 | 24.51 | 3.70 | -4.61 | 10.30 | 41 | 7.60 | 53.61 | 8.37 | -9.32 | 24.52 |
| mean5_small | 44 | -4.75 | 28.45 | 4.29 | -13.40 | 3.90 | 41 | -6.18 | 38.54 | 6.02 | -18.34 | 5.99 |
| mean5_large | 44 | 1.72 | 24.18 | 3.65 | -5.64 | 9.07 | 41 | 6.81 | 53.45 | 8.35 | -10.06 | 23.68 |

**Laboratory 2**

**RNG**

**Table 9. Descriptive statistics of the RNG force data of a small vs. large number for each direction.**

|  | **RNG Left** | | | |  |  | **RNG Right** | | | |  |  |
| --- | --- | --- | --- | --- | --- | --- | --- | --- | --- | --- | --- | --- |
|  |  |  |  |  | 95% Credible Interval | |  |  |  |  | 95% Credible Interval | |
|  | n | Mean | SD | SE | Lower | Upper | n | Mean | SD | SE | Lower | Upper |
| mean1_small | 18 | 6.14 | 7.33 | 1.73 | 2.49 | 9.78 | 16 | -4.84 | 11.79 | 2.95 | -11.13 | 1.44 |
| mean1_large | 18 | 6.05 | 11.23 | 2.65 | 0.47 | 11.64 | 16 | -4.23 | 13.75 | 3.44 | -11.56 | 3.10 |
| mean2_small | 18 | 13.70 | 16.68 | 3.93 | 5.40 | 21.99 | 16 | -0.91 | 18.01 | 4.50 | -10.51 | 8.68 |
| mean2_large | 18 | 13.40 | 20.04 | 4.72 | 3.44 | 23.36 | 16 | -3.80 | 21.63 | 5.41 | -15.33 | 7.73 |
| mean3_small | 18 | 25.13 | 24.91 | 5.87 | 12.75 | 37.52 | 16 | 16.13 | 23.97 | 5.99 | 3.36 | 28.90 |
| mean3_large | 18 | 23.49 | 27.35 | 6.45 | 9.89 | 37.09 | 16 | 12.11 | 26.91 | 6.73 | -2.23 | 26.45 |
| mean4_small | 18 | 30.54 | 35.43 | 8.35 | 12.92 | 48.16 | 16 | 24.40 | 36.90 | 9.23 | 4.74 | 44.06 |
| mean4_large | 18 | 27.17 | 31.82 | 7.50 | 11.35 | 42.99 | 16 | 20.19 | 39.42 | 9.86 | -0.82 | 41.19 |
| mean5_small | 18 | 26.68 | 39.34 | 9.27 | 7.12 | 46.25 | 16 | 32.99 | 43.69 | 10.92 | 9.71 | 56.27 |
| mean5_large | 18 | 28.45 | 40.91 | 9.64 | 8.10 | 48.79 | 16 | 27.45 | 43.66 | 10.92 | 4.18 | 50.71 |
|  | **RNG Down** | | | |  |  | **RNG Up** | | | |  |  |
|  |  |  |  |  | 95% Credible Interval | |  |  |  |  | 95% Credible Interval | |
|  | n | Mean | SD | SE | Lower | Upper | n | Mean | SD | SE | Lower | Upper |
| mean1_small | 18 | 8.89 | 9.66 | 2.28 | 4.08 | 13.69 | 15 | -2.5 | 17.8 | 4.6 | -12.3 | 7.4 |
| mean1_large | 18 | 9.72 | 8.88 | 2.09 | 5.31 | 14.14 | 15 | -6.3 | 16.3 | 4.2 | -15.3 | 2.7 |
| mean2_small | 18 | 18.43 | 19.94 | 4.70 | 8.52 | 28.35 | 15 | -4.6 | 32.7 | 8.5 | -22.7 | 13.6 |
| mean2_large | 18 | 20.70 | 16.95 | 4.00 | 12.26 | 29.13 | 15 | -8.0 | 30.3 | 7.8 | -24.8 | 8.8 |
| mean3_small | 18 | 30.38 | 28.84 | 6.80 | 16.04 | 44.73 | 15 | 5.4 | 44.9 | 11.6 | -19.4 | 30.3 |
| mean3_large | 18 | 33.87 | 26.02 | 6.13 | 20.93 | 46.81 | 15 | 3.4 | 37.8 | 9.8 | -17.6 | 24.3 |
| mean4_small | 18 | 30.28 | 30.62 | 7.22 | 15.05 | 45.50 | 15 | 3.6 | 43.5 | 11.2 | -20.5 | 27.7 |
| mean4_large | 18 | 33.26 | 26.26 | 6.19 | 20.20 | 46.32 | 15 | 6.1 | 37.8 | 9.8 | -14.9 | 27.0 |
| mean5_small | 18 | 26.74 | 28.97 | 6.83 | 12.33 | 41.14 | 15 | 5.7 | 44.7 | 11.5 | -19.0 | 30.5 |
| mean5_large | 18 | 30.86 | 23.24 | 5.48 | 19.30 | 42.42 | 15 | 7.6 | 32.8 | 8.5 | -10.5 | 25.8 |

**Table 10. Descriptive statistics of the SDA force data of a small vs. large operand 1 for each direction.**

|  | **O1 Left** | | | |  |  | **O1 Right** | | | |  |  |
| --- | --- | --- | --- | --- | --- | --- | --- | --- | --- | --- | --- | --- |
|  |  |  |  |  | 95% Credible Interval | |  |  |  |  | 95% Credible Interval | |
|  | n | Mean | SD | SE | Lower | Upper | n | Mean | SD | SE | Lower | Upper |
| mean1_small | 18 | -0.48 | 5.17 | 1.22 | -3.05 | 2.09 | 17 | -0.76 | 9.04 | 2.19 | -5.41 | 3.89 |
| mean1_large | 18 | -0.02 | 4.98 | 1.17 | -2.50 | 2.45 | 17 | -0.86 | 7.94 | 1.93 | -4.94 | 3.22 |
| mean2_small | 18 | -1.16 | 6.60 | 1.55 | -4.44 | 2.12 | 17 | -3.31 | 10.89 | 2.64 | -8.91 | 2.29 |
| mean2_large | 18 | -1.96 | 10.04 | 2.37 | -6.96 | 3.03 | 17 | -3.52 | 13.05 | 3.17 | -10.23 | 3.19 |
| mean3_small | 18 | 3.78 | 10.79 | 2.54 | -1.59 | 9.14 | 17 | -2.18 | 13.55 | 3.29 | -9.15 | 4.79 |
| mean3_large | 18 | 2.07 | 10.36 | 2.44 | -3.08 | 7.22 | 17 | -3.33 | 17.55 | 4.26 | -12.35 | 5.70 |
| mean4_small | 18 | 4.48 | 13.91 | 3.28 | -2.44 | 11.40 | 17 | -0.74 | 16.08 | 3.90 | -9.01 | 7.53 |
| mean4_large | 18 | 2.62 | 9.92 | 2.34 | -2.31 | 7.55 | 17 | -1.52 | 21.40 | 5.19 | -12.52 | 9.49 |
| mean5_small | 18 | 3.07 | 15.35 | 3.62 | -4.57 | 10.70 | 17 | -0.56 | 16.01 | 3.88 | -8.79 | 7.67 |
| mean5_large | 18 | 2.69 | 9.75 | 2.30 | -2.16 | 7.54 | 17 | -1.43 | 22.39 | 5.43 | -12.94 | 10.09 |
|  | **O1 Down** | | | |  |  | **O1 Up** | | | |  |  |
|  |  |  |  |  | 95% Credible Interval | |  |  |  |  | 95% Credible Interval | |
|  | n | Mean | SD | SE | Lower | Upper | n | Mean | SD | SE | Lower | Upper |
| mean1_small | 18 | 3.72 | 8.96 | 2.11 | -0.74 | 8.17 | 16 | -2.81 | 12.37 | 3.09 | -9.40 | 3.78 |
| mean1_large | 18 | 3.76 | 10.43 | 2.46 | -1.43 | 8.95 | 16 | -2.67 | 11.39 | 2.85 | -8.74 | 3.40 |
| mean2_small | 18 | 2.98 | 8.37 | 1.97 | -1.19 | 7.14 | 16 | -6.62 | 12.45 | 3.11 | -13.25 | 0.02 |
| mean2_large | 18 | 1.62 | 12.39 | 2.92 | -4.54 | 7.78 | 16 | -5.56 | 16.50 | 4.13 | -14.36 | 3.23 |
| mean3_small | 18 | 7.76 | 15.36 | 3.62 | 0.12 | 15.40 | 16 | -2.35 | 21.02 | 5.26 | -13.55 | 8.85 |
| mean3_large | 18 | 5.59 | 13.64 | 3.22 | -1.20 | 12.37 | 16 | 1.51 | 13.31 | 3.33 | -5.58 | 8.60 |
| mean4_small | 18 | 9.22 | 15.83 | 3.73 | 1.35 | 17.09 | 16 | 1.00 | 28.70 | 7.18 | -14.30 | 16.29 |
| mean4_large | 18 | 5.95 | 12.51 | 2.95 | -0.27 | 12.17 | 16 | 5.42 | 11.43 | 2.86 | -0.67 | 11.51 |
| mean5_small | 18 | 8.58 | 16.01 | 3.77 | 0.62 | 16.54 | 16 | 3.56 | 30.77 | 7.69 | -12.83 | 19.96 |
| mean5_large | 18 | 4.94 | 13.38 | 3.15 | -1.72 | 11.59 | 16 | 7.55 | 10.32 | 2.58 | 2.06 | 13.05 |

**Table 11. Descriptive statistics of the SDA force data of a plus vs. minus after a small operand 1 for each direction.**

|  | **Operator after small O1 Left** | | | |  |  | **Operator after small O1 Right** | | | |  |  |
| --- | --- | --- | --- | --- | --- | --- | --- | --- | --- | --- | --- | --- |
|  |  |  |  |  | 95% Credible Interval | |  |  |  |  | 95% Credible Interval | |
|  | n | Mean | SD | SE | Lower | Upper | n | Mean | SD | SE | Lower | Upper |
| mean1_minus | 18 | -1.73 | 21.84 | 5.15 | -12.59 | 9.14 | 17 | -4.6 | 38.1 | 9.2 | -24.2 | 15.0 |
| mean1_plus | 18 | 3.89 | 17.39 | 4.10 | -4.76 | 12.54 | 17 | -0.9 | 23.5 | 5.7 | -13.0 | 11.2 |
| mean2_minus | 18 | 2.84 | 24.24 | 5.71 | -9.22 | 14.89 | 17 | -8.3 | 40.2 | 9.7 | -29.0 | 12.4 |
| mean2_plus | 18 | 2.06 | 17.35 | 4.09 | -6.57 | 10.69 | 17 | -2.8 | 24.5 | 5.9 | -15.4 | 9.8 |
| mean3_minus | 18 | 4.36 | 24.06 | 5.67 | -7.61 | 16.32 | 17 | -9.1 | 43.9 | 10.7 | -31.7 | 13.5 |
| mean3_plus | 18 | 4.70 | 16.26 | 3.83 | -3.39 | 12.78 | 17 | -0.8 | 23.2 | 5.6 | -12.8 | 11.1 |
| mean4_minus | 18 | 4.55 | 31.01 | 7.31 | -10.88 | 19.97 | 17 | -12.3 | 47.4 | 11.5 | -36.7 | 12.1 |
| mean4_plus | 18 | 4.42 | 17.98 | 4.24 | -4.52 | 13.36 | 17 | 0.6 | 25.8 | 6.3 | -12.6 | 13.9 |
| mean5_minus | 18 | 6.43 | 33.96 | 8.01 | -10.46 | 23.32 | 17 | -11.9 | 48.2 | 11.7 | -36.7 | 12.9 |
| mean5_plus | 18 | 4.28 | 18.85 | 4.44 | -5.09 | 13.66 | 17 | 0.0 | 26.3 | 6.4 | -13.6 | 13.5 |
|  | **Operator after small O1 Down** | | | |  |  | **Operator after small O1 Up** | | | |  |  |
|  |  |  |  |  | 95% Credible Interval | |  |  |  |  | 95% Credible Interval | |
|  | n | Mean | SD | SE | Lower | Upper | n | Mean | SD | SE | Lower | Upper |
| mean1_minus | 17 | -0.3 | 26.6 | 6.5 | -13.9 | 13.4 | 14 | 1.93 | 30.95 | 8.27 | -15.94 | 19.80 |
| mean1_plus | 17 | 3.5 | 20.9 | 5.1 | -7.3 | 14.2 | 14 | 2.97 | 33.31 | 8.90 | -16.26 | 22.21 |
| mean2_minus | 17 | -2.1 | 27.6 | 6.7 | -16.3 | 12.1 | 14 | -4.05 | 30.04 | 8.03 | -21.39 | 13.30 |
| mean2_plus | 17 | 1.4 | 19.0 | 4.6 | -8.4 | 11.2 | 14 | -0.88 | 30.04 | 8.03 | -18.22 | 16.47 |
| mean3_minus | 17 | -1.3 | 28.5 | 6.9 | -16.0 | 13.4 | 14 | -3.44 | 33.63 | 8.99 | -22.86 | 15.97 |
| mean3_plus | 17 | 1.7 | 17.1 | 4.2 | -7.1 | 10.5 | 14 | -1.39 | 31.42 | 8.40 | -19.53 | 16.75 |
| mean4_minus | 17 | 1.4 | 31.6 | 7.7 | -14.8 | 17.7 | 14 | 3.46 | 37.09 | 9.91 | -17.95 | 24.87 |
| mean4_plus | 17 | 2.2 | 18.1 | 4.4 | -7.1 | 11.6 | 14 | 1.68 | 35.71 | 9.54 | -18.94 | 22.30 |
| mean5_minus | 17 | 1.4 | 32.4 | 7.9 | -15.2 | 18.1 | 14 | 7.34 | 39.97 | 10.68 | -15.74 | 30.42 |
| mean5_plus | 17 | 1.7 | 18.9 | 4.6 | -8.1 | 11.4 | 14 | 3.96 | 34.66 | 9.26 | -16.05 | 23.97 |

**Table 12. Descriptive statistics of the SDA force data of a plus vs. minus after a large operand 1 for each direction.**

|  | **Operator after large O1 Left** | | | |  |  | **Operator after large O1 Right** | | | |  |  |
| --- | --- | --- | --- | --- | --- | --- | --- | --- | --- | --- | --- | --- |
|  |  |  |  |  | 95% Credible Interval | |  |  |  |  | 95% Credible Interval | |
|  | n | Mean | SD | SE | Lower | Upper | n | Mean | SD | SE | Lower | Upper |
| mean1_minus | 18 | 3.94 | 14.20 | 3.35 | -3.12 | 11.00 | 17 | -2.54 | 20.07 | 4.87 | -12.86 | 7.78 |
| mean1_plus | 18 | -1.07 | 17.29 | 4.08 | -9.66 | 7.53 | 17 | -3.36 | 36.23 | 8.79 | -21.98 | 15.27 |
| mean2_minus | 18 | 3.53 | 14.73 | 3.47 | -3.79 | 10.85 | 17 | -3.95 | 21.00 | 5.09 | -14.75 | 6.84 |
| mean2_plus | 18 | -1.40 | 18.78 | 4.43 | -10.74 | 7.94 | 17 | -4.37 | 30.25 | 7.34 | -19.92 | 11.19 |
| mean3_minus | 18 | 5.00 | 15.10 | 3.56 | -2.51 | 12.51 | 17 | -2.61 | 21.58 | 5.24 | -13.71 | 8.49 |
| mean3_plus | 18 | 0.13 | 21.32 | 5.03 | -10.48 | 10.73 | 17 | -5.41 | 30.78 | 7.47 | -21.23 | 10.42 |
| mean4_minus | 18 | 3.02 | 18.15 | 4.28 | -6.01 | 12.04 | 17 | -0.84 | 22.01 | 5.34 | -12.15 | 10.48 |
| mean4_plus | 18 | 2.01 | 23.37 | 5.51 | -9.61 | 13.63 | 17 | -4.65 | 32.34 | 7.84 | -21.28 | 11.98 |
| mean5_minus | 18 | 2.78 | 19.64 | 4.63 | -6.98 | 12.55 | 17 | 0.17 | 23.74 | 5.76 | -12.04 | 12.38 |
| mean5_plus | 18 | 2.61 | 23.00 | 5.42 | -8.82 | 14.05 | 17 | -3.25 | 31.93 | 7.74 | -19.67 | 13.17 |
|  | **Operator after large O1 Down** | | | |  |  | **Operator after large O1 Up** | | | |  |  |
|  |  |  |  |  | 95% Credible Interval | |  |  |  |  | 95% Credible Interval | |
|  | n | Mean | SD | SE | Lower | Upper | n | Mean | SD | SE | Lower | Upper |
| mean1_minus | 17 | 6.20 | 16.52 | 4.01 | -2.29 | 14.70 | 14 | 0.41 | 16.80 | 4.49 | -9.29 | 10.11 |
| mean1_plus | 17 | -5.75 | 24.03 | 5.83 | -18.11 | 6.60 | 14 | -3.01 | 35.30 | 9.43 | -23.39 | 17.37 |
| mean2_minus | 17 | 5.22 | 17.04 | 4.13 | -3.54 | 13.98 | 14 | -4.89 | 17.48 | 4.67 | -14.99 | 5.20 |
| mean2_plus | 17 | -3.03 | 23.44 | 5.69 | -15.08 | 9.02 | 14 | -1.31 | 40.10 | 10.72 | -24.47 | 21.84 |
| mean3_minus | 17 | 5.33 | 18.31 | 4.44 | -4.09 | 14.74 | 14 | -1.11 | 19.10 | 5.10 | -12.14 | 9.92 |
| mean3_plus | 17 | 0.68 | 23.47 | 5.69 | -11.39 | 12.74 | 14 | 10.88 | 40.23 | 10.75 | -12.35 | 34.10 |
| mean4_minus | 17 | 7.60 | 23.12 | 5.61 | -4.29 | 19.49 | 14 | 5.60 | 23.84 | 6.37 | -8.17 | 19.36 |
| mean4_plus | 17 | 3.91 | 25.70 | 6.23 | -9.31 | 17.12 | 14 | 17.99 | 42.76 | 11.43 | -6.70 | 42.68 |
| mean5_minus | 17 | 8.11 | 25.23 | 6.12 | -4.87 | 21.08 | 14 | 6.29 | 25.22 | 6.74 | -8.27 | 20.85 |
| mean5_plus | 17 | 6.27 | 28.05 | 6.80 | -8.16 | 20.69 | 14 | 19.28 | 45.26 | 12.10 | -6.86 | 45.41 |

**Table 13. Descriptive statistics of the SDA force data of a small vs. large operand 2 after a small operand 1 for each direction.**

|  | **O2 after small O1 Left** | | | |  |  | **O2 after small O1 Right** | | | |  |  |
| --- | --- | --- | --- | --- | --- | --- | --- | --- | --- | --- | --- | --- |
|  |  |  |  |  | 95% Credible Interval | |  |  |  |  | 95% Credible Interval | |
|  | n | Mean | SD | SE | Lower | Upper | n | Mean | SD | SE | Lower | Upper |
| mean1_small | 18 | 9.25 | 25.22 | 5.94 | -3.29 | 21.79 | 14 | 3.00 | 36.43 | 9.74 | -18.03 | 24.03 |
| mean1_large | 18 | 3.95 | 35.51 | 8.37 | -13.71 | 21.60 | 14 | -11.20 | 42.31 | 11.31 | -35.64 | 13.23 |
| mean2_small | 18 | 10.43 | 28.26 | 6.66 | -3.62 | 24.49 | 14 | 0.37 | 39.22 | 10.48 | -22.27 | 23.02 |
| mean2_large | 18 | 7.76 | 37.43 | 8.82 | -10.85 | 26.38 | 14 | -11.42 | 48.66 | 13.00 | -39.52 | 16.67 |
| mean3_small | 18 | 13.23 | 26.61 | 6.27 | -0.01 | 26.46 | 14 | 2.81 | 41.21 | 11.02 | -20.99 | 26.61 |
| mean3_large | 18 | 15.06 | 40.43 | 9.53 | -5.04 | 35.17 | 14 | -7.99 | 48.81 | 13.05 | -36.17 | 20.20 |
| mean4_small | 18 | 17.65 | 27.89 | 6.57 | 3.78 | 31.52 | 14 | 1.93 | 44.53 | 11.90 | -23.79 | 27.64 |
| mean4_large | 18 | 19.23 | 44.33 | 10.45 | -2.82 | 41.28 | 14 | -2.89 | 49.39 | 13.20 | -31.41 | 25.62 |
| mean5_small | 18 | 18.75 | 28.67 | 6.76 | 4.49 | 33.01 | 14 | 3.34 | 44.00 | 11.76 | -22.06 | 28.74 |
| mean5_large | 18 | 20.45 | 45.19 | 10.65 | -2.02 | 42.92 | 14 | -1.58 | 49.86 | 13.33 | -30.37 | 27.21 |
|  | **O2 after small O1 Down** | | | |  |  | **O2 after small O1 Up** | | | |  |  |
|  |  |  |  |  | 95% Credible Interval | |  |  |  |  | 95% Credible Interval | |
|  | n | Mean | SD | SE | Lower | Upper | n | Mean | SD | SE | Lower | Upper |
| mean1_small | 16 | 2.62 | 20.86 | 5.22 | -8.50 | 13.74 | 11 | 6.38 | 31.02 | 9.35 | -14.46 | 27.22 |
| mean1_large | 16 | -1.35 | 30.77 | 7.69 | -17.74 | 15.04 | 11 | 3.14 | 47.75 | 14.40 | -28.94 | 35.21 |
| mean2_small | 16 | 2.86 | 19.89 | 4.97 | -7.74 | 13.46 | 11 | 4.40 | 33.07 | 9.97 | -17.82 | 26.61 |
| mean2_large | 16 | -2.52 | 34.60 | 8.65 | -20.96 | 15.92 | 11 | -1.29 | 50.71 | 15.29 | -35.36 | 32.78 |
| mean3_small | 16 | 0.10 | 20.60 | 5.15 | -10.87 | 11.08 | 11 | 7.31 | 36.00 | 10.86 | -16.88 | 31.49 |
| mean3_large | 16 | 0.05 | 36.38 | 9.09 | -19.34 | 19.43 | 11 | 1.92 | 50.09 | 15.10 | -31.73 | 35.57 |
| mean4_small | 16 | 2.01 | 21.89 | 5.47 | -9.66 | 13.67 | 11 | 15.50 | 39.20 | 11.82 | -10.83 | 41.84 |
| mean4_large | 16 | 4.24 | 39.44 | 9.86 | -16.77 | 25.26 | 11 | 3.51 | 44.87 | 13.53 | -26.64 | 33.65 |
| mean5_small | 16 | 3.64 | 23.84 | 5.96 | -9.07 | 16.34 | 11 | 18.58 | 41.68 | 12.57 | -9.42 | 46.58 |
| mean5_large | 16 | 5.45 | 42.00 | 10.50 | -16.93 | 27.82 | 11 | 5.83 | 44.02 | 13.27 | -23.75 | 35.40 |

**Table 14. Descriptive statistics of the SDA force data of a small vs. large operand 2 after a large operand 1 for each direction.**

|  | **O2 after large O1 Left** | | | |  |  | **O2 after large O1 Right** | | | |  |  |
| --- | --- | --- | --- | --- | --- | --- | --- | --- | --- | --- | --- | --- |
|  |  |  |  |  | 95% Credible Interval | |  |  |  |  | 95% Credible Interval | |
|  | n | Mean | SD | SE | Lower | Upper | n | Mean | SD | SE | Lower | Upper |
| mean1_small | 18 | 3.37 | 23.07 | 5.44 | -8.10 | 14.85 | 14 | -5.80 | 29.60 | 7.91 | -22.89 | 11.29 |
| mean1_large | 18 | -2.97 | 35.98 | 8.48 | -20.86 | 14.93 | 14 | 6.74 | 46.37 | 12.39 | -20.04 | 33.51 |
| mean2_small | 18 | 4.51 | 23.88 | 5.63 | -7.37 | 16.39 | 14 | -5.83 | 29.56 | 7.90 | -22.90 | 11.24 |
| mean2_large | 18 | -4.65 | 42.65 | 10.05 | -25.86 | 16.56 | 14 | 8.76 | 43.24 | 11.56 | -16.21 | 33.72 |
| mean3_small | 18 | 9.27 | 23.29 | 5.49 | -2.32 | 20.85 | 14 | -1.22 | 30.92 | 8.26 | -19.07 | 16.63 |
| mean3_large | 18 | 0.94 | 44.28 | 10.44 | -21.08 | 22.96 | 14 | 9.53 | 49.45 | 13.22 | -19.02 | 38.08 |
| mean4_small | 18 | 12.14 | 23.53 | 5.55 | 0.44 | 23.84 | 14 | 5.05 | 33.19 | 8.87 | -14.12 | 24.21 |
| mean4_large | 18 | 7.56 | 45.30 | 10.68 | -14.97 | 30.09 | 14 | 13.27 | 49.13 | 13.13 | -15.10 | 41.64 |
| mean5_small | 18 | 13.08 | 23.79 | 5.61 | 1.25 | 24.91 | 14 | 6.81 | 35.47 | 9.48 | -13.67 | 27.29 |
| mean5_large | 18 | 9.82 | 46.78 | 11.03 | -13.44 | 33.08 | 14 | 19.25 | 52.03 | 13.91 | -10.79 | 49.30 |
|  | **O2 after large O1 Down** | | | |  |  | **O2 after large O1 Up** | | | |  |  |
|  |  |  |  |  | 95% Credible Interval | |  |  |  |  | 95% Credible Interval | |
|  | n | Mean | SD | SE | Lower | Upper | n | Mean | SD | SE | Lower | Upper |
| mean1_small | 16 | 8.27 | 23.24 | 5.81 | -4.11 | 20.66 | 11 | 7.38 | 22.60 | 6.81 | -7.80 | 22.56 |
| mean1_large | 16 | -8.92 | 38.65 | 9.66 | -29.52 | 11.68 | 11 | 5.37 | 40.65 | 12.26 | -21.94 | 32.68 |
| mean2_small | 16 | 6.78 | 25.59 | 6.40 | -6.85 | 20.42 | 11 | 9.81 | 24.91 | 7.51 | -6.92 | 26.55 |
| mean2_large | 16 | -6.49 | 40.43 | 10.11 | -28.03 | 15.05 | 11 | 2.06 | 37.08 | 11.18 | -22.86 | 26.97 |
| mean3_small | 16 | 6.23 | 26.25 | 6.56 | -7.76 | 20.21 | 11 | 14.42 | 30.43 | 9.18 | -6.03 | 34.86 |
| mean3_large | 16 | -2.21 | 39.59 | 9.90 | -23.31 | 18.89 | 11 | 2.69 | 36.49 | 11.00 | -21.83 | 27.20 |
| mean4_small | 16 | 7.96 | 28.98 | 7.25 | -7.49 | 23.40 | 11 | 20.09 | 29.59 | 8.92 | 0.21 | 39.97 |
| mean4_large | 16 | -0.72 | 39.54 | 9.88 | -21.78 | 20.35 | 11 | 7.16 | 36.06 | 10.87 | -17.06 | 31.39 |
| mean5_small | 16 | 9.44 | 30.76 | 7.69 | -6.95 | 25.84 | 11 | 23.18 | 28.70 | 8.65 | 3.90 | 42.46 |
| mean5_large | 16 | 0.05 | 39.79 | 9.95 | -21.16 | 21.25 | 11 | 11.67 | 36.89 | 11.12 | -13.12 | 36.45 |

**Table 15. Descriptive statistics of the SDA force data of a small vs. large answer after a small operand 1 for each direction.**

|  | **Answer after small O1 Left** | | | |  |  | **Answer after small O1 Right** | | | |  |  |
| --- | --- | --- | --- | --- | --- | --- | --- | --- | --- | --- | --- | --- |
|  |  |  |  |  | 95% Credible Interval | |  |  |  |  | 95% Credible Interval | |
|  | n | Mean | SD | SE | Lower | Upper | n | Mean | SD | SE | Lower | Upper |
| mean1_small | 18 | 32.98 | 41.58 | 9.80 | 12.31 | 53.66 | 14 | 9.85 | 59.38 | 15.87 | -24.43 | 44.14 |
| mean1_large | 18 | 10.54 | 34.00 | 8.01 | -6.37 | 27.45 | 14 | 15.38 | 62.48 | 16.70 | -20.70 | 51.45 |
| mean2_small | 18 | 33.47 | 37.20 | 8.77 | 14.97 | 51.97 | 14 | -1.32 | 61.99 | 16.57 | -37.11 | 34.47 |
| mean2_large | 18 | 11.36 | 33.77 | 7.96 | -5.44 | 28.15 | 14 | 2.87 | 61.10 | 16.33 | -32.41 | 38.15 |
| mean3_small | 18 | 36.75 | 34.81 | 8.20 | 19.44 | 54.06 | 14 | 1.16 | 63.54 | 16.98 | -35.53 | 37.85 |
| mean3_large | 18 | 18.41 | 33.28 | 7.84 | 1.87 | 34.96 | 14 | 7.24 | 57.32 | 15.32 | -25.85 | 40.34 |
| mean4_small | 18 | 38.29 | 35.54 | 8.38 | 20.62 | 55.96 | 14 | 4.76 | 72.49 | 19.37 | -37.10 | 46.61 |
| mean4_large | 18 | 16.36 | 33.88 | 7.99 | -0.49 | 33.20 | 14 | 7.59 | 57.03 | 15.24 | -25.34 | 40.52 |
| mean5_small | 18 | 36.98 | 36.20 | 8.53 | 18.98 | 54.98 | 14 | 5.28 | 74.10 | 19.80 | -37.51 | 48.06 |
| mean5_large | 18 | 13.02 | 35.02 | 8.26 | -4.40 | 30.44 | 14 | 7.30 | 58.07 | 15.52 | -26.24 | 40.83 |
|  | **Answer after small O1 Down** | | | |  |  | **Answer after small O1 Up** | | | |  |  |
|  |  |  |  |  | 95% Credible Interval | |  |  |  |  | 95% Credible Interval | |
|  | n | Mean | SD | SE | Lower | Upper | n | Mean | SD | SE | Lower | Upper |
| mean1_small | 16 | -2.96 | 45.75 | 11.44 | -27.34 | 21.42 | 5 | -6.70 | 56.12 | 25.10 | -76.39 | 62.98 |
| mean1_large | 16 | 6.66 | 27.74 | 6.93 | -8.12 | 21.44 | 5 | 27.36 | 69.82 | 31.22 | -59.33 | 114.05 |
| mean2_small | 16 | -3.56 | 49.01 | 12.25 | -29.68 | 22.56 | 5 | -12.06 | 50.99 | 22.81 | -75.37 | 51.26 |
| mean2_large | 16 | 0.10 | 30.45 | 7.61 | -16.13 | 16.32 | 5 | 26.43 | 65.43 | 29.26 | -54.81 | 107.67 |
| mean3_small | 16 | -2.48 | 50.75 | 12.69 | -29.52 | 24.57 | 5 | -14.65 | 54.02 | 24.16 | -81.73 | 52.44 |
| mean3_large | 16 | -2.52 | 33.63 | 8.41 | -20.44 | 15.40 | 5 | 26.28 | 71.64 | 32.04 | -62.67 | 115.24 |
| mean4_small | 16 | 0.33 | 56.07 | 14.02 | -29.55 | 30.20 | 5 | -17.12 | 58.97 | 26.37 | -90.34 | 56.09 |
| mean4_large | 16 | -4.50 | 32.63 | 8.16 | -21.89 | 12.89 | 5 | 23.41 | 82.30 | 36.81 | -78.78 | 125.60 |
| mean5_small | 16 | 0.91 | 58.27 | 14.57 | -30.14 | 31.96 | 5 | -19.59 | 50.53 | 22.60 | -82.33 | 43.16 |
| mean5_large | 16 | -4.48 | 31.21 | 7.80 | -21.11 | 12.15 | 5 | 21.29 | 84.51 | 37.79 | -83.65 | 126.22 |

**Table 16. Descriptive statistics of the SDA force data of a small vs. large answer after a large operand 1 for each direction.**

|  | **Answer after large O1 Left** | | | |  |  | **Answer after large O1 Right** | | | |  |  |
| --- | --- | --- | --- | --- | --- | --- | --- | --- | --- | --- | --- | --- |
|  |  |  |  |  | 95% Credible Interval | |  |  |  |  | 95% Credible Interval | |
|  | n | Mean | SD | SE | Lower | Upper | n | Mean | SD | SE | Lower | Upper |
| mean1_small | 18 | 24.15 | 35.66 | 8.41 | 6.41 | 41.88 | 14 | 34.51 | 68.83 | 18.40 | -5.23 | 74.26 |
| mean1_large | 18 | 27.68 | 46.34 | 10.92 | 4.64 | 50.73 | 14 | -11.22 | 36.49 | 9.75 | -32.29 | 9.85 |
| mean2_small | 18 | 27.11 | 35.55 | 8.38 | 9.43 | 44.78 | 14 | 25.53 | 64.94 | 17.36 | -11.97 | 63.02 |
| mean2_large | 18 | 28.62 | 45.54 | 10.73 | 5.97 | 51.27 | 14 | -22.04 | 39.80 | 10.64 | -45.02 | 0.94 |
| mean3_small | 18 | 31.04 | 37.76 | 8.90 | 12.26 | 49.82 | 14 | 27.53 | 68.83 | 18.40 | -12.22 | 67.27 |
| mean3_large | 18 | 32.53 | 42.21 | 9.95 | 11.54 | 53.51 | 14 | -29.27 | 48.45 | 12.95 | -57.25 | -1.30 |
| mean4_small | 18 | 28.82 | 36.89 | 8.70 | 10.48 | 47.17 | 14 | 32.26 | 71.17 | 19.02 | -8.84 | 73.35 |
| mean4_large | 18 | 32.33 | 37.31 | 8.79 | 13.77 | 50.88 | 14 | -27.31 | 50.10 | 13.39 | -56.23 | 1.62 |
| mean5_small | 18 | 28.69 | 35.20 | 8.30 | 11.19 | 46.20 | 14 | 31.40 | 73.51 | 19.65 | -11.04 | 73.84 |
| mean5_large | 18 | 29.33 | 35.16 | 8.29 | 11.85 | 46.82 | 14 | -24.26 | 48.25 | 12.90 | -52.12 | 3.60 |
|  | **Answer after large O1 Down** | | | |  |  | **Answer after large O1 Up** | | | |  |  |
|  |  |  |  |  | 95% Credible Interval | |  |  |  |  | 95% Credible Interval | |
|  | n | Mean | SD | SE | Lower | Upper | n | Mean | SD | SE | Lower | Upper |
| mean1_small | 16 | 4.4 | 42.3 | 10.6 | -18.2 | 26.9 | 5 | 40.5 | 52.8 | 23.6 | -25.0 | 106.0 |
| mean1_large | 16 | -1.4 | 44.0 | 11.0 | -24.9 | 22.1 | 5 | -3.1 | 58.8 | 26.3 | -76.1 | 69.9 |
| mean2_small | 16 | 3.9 | 41.1 | 10.3 | -18.0 | 25.9 | 5 | 34.1 | 52.2 | 23.3 | -30.7 | 98.8 |
| mean2_large | 16 | -4.8 | 44.6 | 11.1 | -28.5 | 19.0 | 5 | -5.6 | 61.6 | 27.5 | -82.1 | 70.8 |
| mean3_small | 16 | 3.8 | 42.7 | 10.7 | -19.0 | 26.5 | 5 | 33.1 | 50.3 | 22.5 | -29.4 | 95.5 |
| mean3_large | 16 | -6.8 | 45.6 | 11.4 | -31.1 | 17.5 | 5 | -2.6 | 61.9 | 27.7 | -79.5 | 74.3 |
| mean4_small | 16 | 5.1 | 35.4 | 8.9 | -13.8 | 24.0 | 5 | 40.3 | 44.6 | 19.9 | -15.0 | 95.7 |
| mean4_large | 16 | -6.7 | 46.5 | 11.6 | -31.5 | 18.1 | 5 | -9.6 | 55.7 | 24.9 | -78.7 | 59.5 |
| mean5_small | 16 | 4.7 | 31.3 | 7.8 | -12.0 | 21.4 | 5 | 41.9 | 43.0 | 19.2 | -11.5 | 95.3 |
| mean5_large | 16 | -5.0 | 49.3 | 12.3 | -31.3 | 21.2 | 5 | -13.1 | 49.5 | 22.1 | -74.5 | 48.3 |
