# Supplementary material for: How to not induce SNAs: The insufficiency of directional force
Source: PLoS One. 2023 Jun 29;18(6):e0288038. doi: 10.1371/journal.pone.0288038 (PMC10309995; doi:10.1371/journal.pone.0288038)
Supplement: Attachment — Submitted filename: Response to reviewers_Revision2.pdf [file pone.0288038.s008.pdf]

Dear Dr. Tessari,

We are here submitting our second revision of the manuscript „How to not induce SNAs: the insufficiency of covert directional movement.” to PlosOne. Due to reviewers’ feedback, it is now retitled to “**How to not induce SNAs: the insufficiency of directional force**”.

We thank both reviewers for their valuable comments, and we have responded to all their suggestions/comments. This letter (below) describes how we have made the changes in response to each of the reviewers’ comments. All relevant changes are tracked in the revised manuscript. For this letter, all indicated lines refer to the tracked manuscript (markup-all). On the last page, we also included the updated reference list indicating the changes made throughout the review process.

Due to the helpful feedback from the reviewers, we have significantly improved the revised manuscript, and we think it can now have a substantial impact on the field.

Feel free to contact me if you need further information and/or clarification(s) on any changes we have made.

We look forward to hearing from you soon.

Yours Sincerely,

Katharina Kühne (kkuehne@uni-potsdam.de),

on behalf of all authors.

Reviewer #1:

The manuscript has been improved in many ways. Still, I found a number of inadequacies and also have some suggestions for further improvement. I will mention them in the same order as they appear in the manuscript.

- Title: I am not convinced by the label "covert movement". In my opinion, a movement is covert if it is planned and mentally simulated, but without it reaching the muscles. In this study the planned action does reach the muscles, and the participant does not plan to perform any movement, just a permanent pressure. It would be better to use a different label. "Isometric force" maybe a good alternative, although when I first read it I had difficulty to parse it. Maybe "static directional pressure" or some other option.

Reply:

Thanks to both reviewers for pointing this out. We have now completely removed the words "overt" and "covert" from the manuscript as both reviewers made similar points regarding the wording and how it led to confusion. We now use "isometric force" and "isometric directional force" and already applied them in the title. Additionally, we have reformulated the sentence with the aim of the study to make the idea more accessible before introducing "isometric directional force". We have also contrasted movement through space with our isometric directional force manipulation early in the Discussion.

We have renamed the manuscript to "**How to not induce SNAs: the insufficiency of directional force**".

In lines 324-326, it now reads:

"Will directional but non-spatial movement along the horizontal and vertical axes, thus <u>pressing into a direction of left, right, up, or down,</u> elicit SNAs?".

In lines 328-338, we follow up with:

"We asked participants to press against a sensor surface, thus recording their isometric force production. Such isometric force was exerted constantly and continuously on the sensor surface in a direction on the horizontal (left or right) and the vertical (up or down) axes during two tasks. Hereby, we utilized the same force sensors as Miklashevsky et al. [41] but altered the paradigm to record the production of isometric force by one-handed presses into a direction instead of grip force (from here on described as "isometric directional force")."

In lines 695-699, it now reads:

"Notable, our isometric directional force manipulation did not include a movement through space. Instead, isometric directional force utilized a subtler approach of directionally applied pressure attempting to elicit spatial codes.

- line 54: "produces" is not adequate. Options: "supports", "suggests"...

Reply:

Corrected. In lines 54-56, it now reads:

“The reaction time paradigm is widely accepted and provides evidence for the association of smaller numbers with left space and larger numbers with right space [4].”

- lines 72-80 (polarity correspondence account): It would be important to cite the work by Santiago and Lakens (2015) that failed to support the polarity correspondence account. In general, I don’t think there is much point of describing and discussing all these theoretical approaches in the introduction of the paper, as the data are not going to be relevant to any of them and they are not taken up again in the discussion.

Reply:

We have referenced the Santiago and Lakens (2015) paper to address existing counterevidence for the polarity correspondence account. In lines 80-82, it now reads:

“Therefore, SNARC would not be limited to the horizontal axis (but see [11] for empirical evidence against the polarity correspondence account).”

Regarding the Introduction. The theoretical approaches discussed in the Introduction seem important to keep allowing us to introduce the context in which the experiment is based on the existing literature. This approach reflects our planning in the methodology and facilitates the comprehension of our study for the readers. Thus, we tried to be concise in our Introduction but respectfully decline the request to eliminate our theoretical context. Moreover, to establish better theoretical coherence between Introduction and Discussion, we now briefly discuss that the theories may need to be extended to allow predictions in cases of an absent movement (see lines 725-735).

References:

Santiago, J., & Lakens, D. (2015). Can conceptual congruency effects between number, time, and space be accounted for by polarity correspondence? Acta Psychologica, 156, 179–191. https://doi.org/10.1016/j.actpsy.2014.09.016

- line 84: not all embodied approaches presume that the relation between the abstract and the concrete domain must be bidirectional. Conceptual metaphor theory is an embodied theory and it suggests an asymmetrical relation. Moreover, not all abstract concepts are the same: some abstract concepts seem to be of a different kind to numbers, being more based on interoceptive experiences and language (see the recent review by Borghi, Shaki, and Fischer, 2022).

Reply:

Thank you for the suggestions. We now acknowledge the asymmetrical relation of numbers and space. In lines 102-105, it now reads:

“Notably, there is an asymmetrical relationship between space and numbers, in which the understanding of space is more fundamental. The meaning of numbers is often based on the experiences of space while this is not necessarily the case vice versa [12,13].

Additionally, we have integrated the review by Borghi, Shaki, and Fischer (2022) into an existing section where we compared the mechanisms behind the vertical and the horizontal SNAs (lines 249-268).

Concretely, in lines 261-265, it now reads:

“These linguistic practices that describe vertical SNAs differ in their contribution to the embodiment of numbers compared to the horizontal SNAs that seem to rely more on sensorimotor experiences such as finger counting. Moreover, situated factors like interoceptive signals play an additional role in the perception and production of numbers that also might contribute to SNAs ([50]; for review see [51]).

References:

Belli, F., Felisatti, A., & Fischer, M. H. (2021). “BreaThink”: breathing affects production and perception of quantities. *Experimental Brain Research*, *239*(8), 2489–2499. https://doi.org/10.1007/s00221-021-06147-z

Borghi, A. M., Shaki, S., & Fischer, M. H. (2023). Abstract concepts: external influences, internal constraints, and methodological issues. *Psychological Research*, *86*(8), 2370–2388. https://doi.org/10.1007/s00426-022-01698-4

- line 126: “Become associated with space” does not seem to me to be the best way to put it. It is clear that numbers have an association with space, and this association is built because of the accummulation of experiences. I think what the authors mean is that they want to study the conditions under which the association between numbers and space is manifested in behavior.

<u>Reply:</u>

Corrected. In lines 133-135, it now reads:

“The goal of the present study is to test the boundary conditions under which the association of numbers and space is manifested in behavior.”.

- line 131: the authors claim that the “common ingredient” of studies showing SNAs is physical space. However, this is not always the case. For Shaki and Fischer (2018) the key ingredient is that either number or space is part of the explicit definition of the task.

<u>Reply:</u>

Thank you for pointing this out. We have now removed the term “physical space” and corrected it to “spatial information”. Spatial information is a broader term that covers the response space, presentation space, and cueing.

In lines 140-145, it now reads:

“By this rationale, the common ingredient for most studies seems to be the activation of spatial information, namely under the experimental conditions of response space (lateral responses), presentation space (lateralized stimuli), or spatial cueing (arrows and/or instructions) during stimulus presentation (see Fig 1 for a visual overview of prototypical laboratory set-ups).”

- line 150: under these conditions, Shaki and Fischer (2018) DID find a vertical SNA in one experiment (but none in the other experiment).

<u>Reply:</u>

This is correct; however, the reviewer seems to have mixed up the order of the references. Line 150 (162-163 in the revised marked manuscript) refers to the study by Pinto et al. (2021; reference number 30) where no SNAs were found in experiment 2. Reference number 31(lines 160-161) is for Shaki and Fischer (2018) only describing the experimental design with no description of the result.

- line 181: the authors try and explain the results by Miklashevsky by having spatial information in the response space. However, other studies such as Shaki and Fischer (2018) did not have any spatial information in the response space and found lateral SNAs in magnitud comparison and a vertical SNA in parity judgement in one out of two experiments. Moreover, there are a number of studies that just present a number and find SNAs in the pattern of eye movements over a blank screen. The authors should try and give a coherent explanation of the whole pattern of findings currently available.

<u>Reply:</u>

Thank you for the comment. The studies that we refer to in the Introduction point to the inconsistency of the literature and how we use it to motivate our own research. For this, we chose the best-fitting examples. However, based on the latter comments of the reviewer, we have also extended the literature by integrating some new studies suggested by the reviewer (lines 226-248). Together, these studies motivate our research and we do not think that adding even more examples would benefit the paper. We think that from lines 244-246 we can already draw the necessary conclusion:

“Together, the above-presented studies point to the conclusion that both, number magnitude and spatial information must be coactivated to produce reliable SNAs [30,31,48,49].”.

References to the additional studies included:

Pinto, M., Pellegrino, M., Marson, F., Lasaponara, S., Cestari, V., D'Onofrio, M., & Doricchi, F. (2021). How to trigger and keep stable directional Space–Number Associations (SNAs). Cortex, 134, 253–264. https://doi.org/10.1016/j.cortex.2020.10.020

Pinto, M., Pellegrino, M., Marson, F., Lasaponara, S., & Doricchi, F. (2019). Reconstructing the origins of the space-number association: spatial and number-magnitude codes must be used jointly to elicit spatially organised mental number lines. Cognition, 190, 143–156. https://doi.org/10.1016/j.cognition.2019.04.032

- line 214: the cite to Pinto [24] does not seem to be correct, as it refers to the study with neglect patients. Actually, it would be important to include all the studies by Pinto (2019 a y b, and 2021) into the set of findings that the authors try and integrate into their review of the literature.

Reply:

Thank you for pointing this out. We have verified the citation (Pinto et al. 2021) and can confirm its accuracy (reference number 30 in the revised manuscript). This was a single but important citation showing an experimental design of a centralized presentation and response space. Based on your suggestions and for the interested readers, we have now referenced other studies by Pinto that fulfill the same criteria and further strengthen our argument. In lines 233-237, it now reads:

"The authors' interpretation was that the numbers will only spatially align across the mental number line when both, number magnitude is activated and spatial response codes are present (for experimental designs that also centralized the presentation and response space see [48,49])."

References:

Pinto, M., Pellegrino, M., Lasaponara, S., Scozia, G., D'Onofrio, M., Raffa, G., Nigro, S., Arnaud, C. R., Tomaiuolo, F., & Doricchi, F. (2021). Number space is made by response space: Evidence from left spatial neglect. Neuropsychologia, 154. https://doi.org/10.1016/j.neuropsychologia.2021.107773

Pinto, M., Pellegrino, M., Marson, F., Lasaponara, S., Cestari, V., D'Onofrio, M., & Doricchi, F. (2021). How to trigger and keep stable directional Space–Number Associations (SNAs). Cortex, 134, 253–264. https://doi.org/10.1016/j.cortex.2020.10.020

Pinto, M., Pellegrino, M., Marson, F., Lasaponara, S., & Doricchi, F. (2019). Reconstructing the origins of the space-number association: spatial and number-magnitude codes must be used jointly to elicit spatially organised mental number lines. Cognition, 190, 143–156. https://doi.org/10.1016/j.cognition.2019.04.032

- line 221: the conclusion that follows from Pinto's studies is not that left and right spatial codes are necessary for SNAs. Instead, it is that both numbers and space must be present AND linked in the definition of the task.

<u>Reply:</u>

Thank you for pointing this out. We have now added multiple sentences to incorporate this.

In lines 233-237, it now reads:

"The authors' interpretation was that the numbers will only spatially align across the mental number line when both, number magnitude is activated and spatial response codes are present (for experimental designs that also centralized the presentation and response space see [48,49])."

In lines, 244-246:

"Together, the above-presented studies point to the conclusion that in which both, number magnitude and spatial information must be coactivated to produce reliable SNAs [30,31,48,49]."

- line 225-226: incorrect. Shaki and Fischer (2018) found absolutely no horizontal SNARC in the parity task.

<u>Reply:</u>

Corrected (line 241).

- lines 228-230: not completely correct: the vertical SNARC in the parity task was found in one experiment but not in the other.

<u>Reply:</u>

Thank you for the comment. We agree that our report was not complete as we do not present the full findings of the experiment because this goes beyond the scope of the paper. However, based on the reviewer's other suggestions the section leads to the conclusion we needed to motivate our research.

In lines 244-246, it now reads:

"Together, the above-presented studies point to the conclusion that both, number magnitude and spatial information must be coactivated to produce reliable SNAs [30,31,48,49].".

- line 293: it is not correct to say that spatial cueing induces vertical but not horizontal SNAs with centralized stimuli and responses (see the last two comments).

Reply:

Corrected. In lines 319-321, it now reads: “Third, cognitive spatial cueing induces stronger vertical than horizontal SNAs during centralized stimulus presentation and responses [31].”

Additionally, we have corrected a sentence in lines 249-253 that now reads:

“One possible explanation for why vertical SNAs remained stronger than horizontal SNAs could be the hierarchical nature of body-related knowledge representations involved in the task, according to which different mechanisms induce different types of SNAs, referred to as grounded, embodied, or situated cognition [29].”.

- line 306: it is asserted that the present study uses “the same isometric force paradigm as Miklashevsky”. Even though the same sensor is used, the fact that Miklashevsky used a two-handed precision grip is a potentially important difference in paradigm (as discussed by the authors at the end of the paper). Therefore, it is misleading to say here that the paradigm in the two studies was the same.

Reply:

We appreciate your helpful suggestion and corrected the sentence. In lines 334-338, it now reads:

“Hereby, we utilized the same force sensors as Miklashevsky et al. [41] but altered the paradigm to record the production of isometric force by one-handed presses into a direction instead of grip force (from here on described as “isometric directional force”).“

- line 395: this is two designs, not one.

- line 399-400: exchange H1 and H2.

Reply to both points:

Thank you for pointing this out. In lines 441-450, now reads:

“In order to test the concept-motor interactions of H1 and H2 with the same task, we have utilized two within-subject designs. Both designs utilized one within-subject factor being the force direction (two levels each: left/right on a horizontal axis or up/down on the vertical axis). To test H1, number magnitude constituted the dependent variable with two magnitude levels: small and large. To test H2, number magnitude constituted the independent variable with two magnitude levels: small and large while continuous isometric force was the dependent variable.”.

Regarding the second point, we cannot exchange H1 and H2 as this would be incorrect.

- line 438: this is two designs, not one.

In lines 490-494, we describe the design of the SDA task. This task does not test the bi-directionality of motor-concept interactions but only the effect of number magnitudes on force magnitudes. Therefore, it is one design.

- lines 474-477: the time windows overlap.

<u>Reply:</u>

The time windows were constructed manually after the uninformative cluster permutation analysis of both tasks. They were constructed to capture mainly the effects in the time windows of 100-250 ms. However, we covered the duration until the end of the trial to explore the data in more detail. Mathematically, the time windows had to overlap during the last fragment of the SDA trials. In lines 480-487, we describe the full reasoning. Concretely, in lines 485-487 it reads:

"These time-windows were constructed to capture mainly the effects in the time-windows of 100-250 ms that are associated with semantic activation after a critical stimulus [72].".

- line 654: as I have pointed out above, "spatial information seems to be deeply rooted into SNAs assessment" fails to take into account Pinto's studies.

<u>Reply:</u>

Thank you for the comment. Earlier, we have acknowledged the position by Pinto et al. (2019, 2021a,2021b) in lines 244-246. However, in that specific paragraph we emphasize on the role of spatial information in the movement itself.

We have rewritten the section to make the point clear. In lines 719-725, it now reads:

"Our finding is in contrast to other RNG studies that found SNAs along the horizontal and vertical axes by using either active movement ([59]; see also [56] for RNG across the horizontal axis; for review see [28]) or passive movement through space [60]. Therefore, it is likely that activating a spatial code in the response space, requires a movement with a spatial component, such as movement through space or movement allocated in space (e.g., contacting spatially aligned response buttons), to create SNAs.".

- line 705: I can't see how the argument in the prior lines (703-704) "fits the reasoning of "Absence of evidence is not evidence of absence". Bayesian statistics are indeed able to quantify evidence for absence.

<u>Reply:</u>

Thank you for pointing this out. We have removed the sentence (lines 787-788).

- line 738: again, the authors claim that Miklashevsky used the same method as in the present study. Although they also measured isometric force, the differences in methods are substantial.

Reply:

Thank you for pointing this out. We have removed the comparison to our study. Further differences from our study to Miklashevksy's are discussed in the Limitations section. In lines 821-823, it now reads:

"Second, as stated in the introduction, Miklashevsky et al. [41] found SNAs in numerical tasks during bimanual and passive isometric force recordings.".

- line 813-814: From the present study it does not follow that "lateralized spatial information in the design space plays a crucial role in eliciting SNAs". This is because in the present design there is lateralized spatial information, as the participants are instructed to press the sensor in lateral (and vertical) directions, and because, in general, the study cannot conclude anything about what is essential for SNAs to arise. It can only conclude that directional static pressures are not able by themselves to make SNAs arise.

Reply:

Thank you for pointing this out. We have now changed the concluding sentence in lines 900-901 that now reads as:

"We conclude that applying pressure into the direction of left, right, up, and down does not suffice in eliciting SNAs along the horizontal and the vertical axes.".

Reviewer #2:

The authors responded admirably to the feedback they received. I see no reason that the paper should not be accepted at this point.

Still, I want to comment on one issue. Previously, I'd found myself confused about the key distinction in the paper between 'overt' and 'covert' movement. The authors' reply about this point left me more confused, I think. Ordinarily I wouldn't care so much about this difference, but it is central to the paper. I'm not sure that (1) this distinction is so clear-cut as the authors imply, that (2) the distinction here is one that maps onto how most people would naturally think of the difference, nor that (3) this distinction is the relevant one. In addition, I felt that grouping all three of the studies I mentioned as 'overt' was very surprising to me. I genuinely wouldn't have expected that. For that reason, I was hoping the authors would have said slightly more in the paper about exactly what things are and are not overt, referring to more specific

examples like these. (I found the information in Figure 1 to be valuable but hard to fully process, even with extensive knowledge of these studies; a non-expert might have even more trouble.)

I don't find this disqualifying, however, because the results can be understood and interpreted without respect to that distinction. For this reason, I only wish to suggest that the authors should once more reconsider whether this language is apt, or whether it is being explained in the best way. Maybe it is; I'm not sure. It stood out to me, so I wanted to make a note of it.

Reply:

Thanks to both reviewers for pointing this out. We have now completely removed the words “overt” and “covert” from the manuscript as both reviewers made similar points regarding the wording and how it led to confusion. We now use “isometric force” and “isometric directional force” and already applied it in the title. Additionally, we have reformulated the sentence with the aim of the study to make the idea more accessible before introducing” isometric directional force”. We have also contrasted movement through space with our isometric directional force manipulation early in the discussion.

We have renamed the manuscript to “**How to not induce SNAs: the insufficiency of directional force**”.

In lines 324-326, it now reads:

“Will directional but non-spatial movement along the horizontal and vertical axes, thus pressing into a direction of left, right, up, or down, elicit SNAs?”.

In lines 328-338, we follow up with:

“We asked participants to press against a sensor surface, thus recording their isometric force production. Such isometric force was exerted constantly and continuously on the sensor surface in a direction on the horizontal (left or right) and the vertical (up or down) axes during two tasks. Hereby, we utilized the same force sensors as Miklashevsky et al. [41] but altered the paradigm to record the production of isometric force by one-handed presses into a direction instead of grip force (from here on described as “isometric directional force”). ”.

In lines 695-699, it now reads:

“Notable, our isometric directional force manipulation did not include a movement through space. Instead, isometric directional force utilized a subtler approach of directionally applied pressure attempting to elicit spatial codes.

Changes to the reference list

The reference list changed from the original submission to the current version. Beneath, the updated reference list is displayed. Red-coloured references are new additions while the orange references were removed from the reference list. The order has changed due to additions, references deletions, and the altered referencing order.

Reference list changes from the first submission to the current version:

1. Dehaene S, Brannon EM. Space, time, and number: a Kantian research program. 2010. doi:10.1016/j.tics.2010.09.009

2. Dehaene S, Bossini S, Giraux P. The mental representation of parity and number magnitude. J Exp Psychol Gen. 1993;122: 371–396. doi:10.1037/0096-3445.122.3.371

3. Fischer MH, Shaki S. Spatial associations in numerical cognition-From single digits to arithmetic. Quarterly Journal of Experimental Psychology. Psychology Press Ltd; 2014. pp. 1461–1483. doi:10.1080/17470218.2014.927515

4. Wood GM de O, Willmes K, Nuerk H, Fischer MH. On the Cognitive Link between Space and Number: A Meta-Analysis of the SNARC Effect. Psychol Sci Q. 2008 [cited 19 Aug 2021]. Available: https://psycnet.apa.org/record/2009-00781-003

5. De Hevia MD, Veggiotti L, Streri A, Bonn CD. At Birth, Humans Associate “Few” with Left and “Many” with Right. Curr Biol. 2017;27: 3879-3884.e2. doi:10.1016/j.cub.2017.11.024

6. Rugani R, Lunghi M, Di Giorgio E, Regolin L, Dalla B, Vallortigara G, et al. A mental number line in human newborns. bioRxiv. 2017; 159335. doi:10.1101/159335

7. Felisatti A, Laubrock J, Shaki S, Fischer MH. A biological foundation for spatial–numerical associations: The brain’s asymmetric frequency tuning. Ann N Y Acad Sci. 2020;1477: 44–53. doi:10.1111/nyas.14418

8. Abrahamse E, Van Dijck JP, Fias W. How does working memory enable number-induced spatial biases? Front Psychol. 2016;7. doi:10.3389/fpsyg.2016.00977

9. Proctor RW, Cho YS. Polarity correspondence: A general principle for performance of speeded binary classification tasks. Psychol Bull. 2006;132: 416–442. doi:10.1037/0033-2909.132.3.416

10. Proctor RW, Xiong A. Polarity Correspondence as a General Compatibility Principle. Curr Dir Psychol Sci. 2015;24: 446–451. doi:10.1177/0963721415607305

11. Santiago J, Lakens D. Can conceptual congruency effects between number, time, and space be accounted for by polarity correspondence? Acta Psychol (Amst). 2015;156: 179–191. doi:10.1016/j.actpsy.2014.09.016

~~11~~.12. Lakoff G, Johnson M. Conceptual Metaphor in Everyday Language. J Philos. 1980;77: 453. doi:10.2307/2025464

~~12.~~ 13. Lakoff G, Núñez RE. Where Mathematics Comes From How: the Embodied Mind Brings Mathematics into Being. New York: Basic Books; 2000.

~~13.~~ 14. Fischer MH. Finger counting habits modulate spatial-numerical associations. Cortex. 2008;44. doi:10.1016/j.cortex.2007.08.004

~~14.~~ 15. Gunderson EA, Spaepen E, Gibson D, Goldin-Meadow S, Levine SC. Gesture as a window onto children's number knowledge. Cognition. 2015;144: 14–28. doi:10.1016/j.cognition.2015.07.008

~~15.~~ 16. Barrocas R, Roesch S, Gawrilow C, Moeller K. Putting a Finger on Numerical Development – Reviewing the Contributions of Kindergarten Finger Gnosis and Fine Motor Skills to Numerical Abilities. Front Psychol. 2020;11. doi:10.3389/fpsyg.2020.01012

~~16.~~17. Michirev A, Musculus L, Raab M. A Developmental Embodied Choice Perspective Explains the Development of Numerical Choices. Front Psychol. 2021;12: 3261. doi:10.3389/fpsyg.2021.694750

~~17.~~ 18. Overmann KA. Finger-counting in the Upper Palaeolithic. Rock Art Res. 2021;31: 63–80. doi:10.31235/osf.io/wgbe5

~~18~~. 19. Fischer MH, Brugger P. When digits help digits: Spatial-numerical associations point to finger counting as prime example of embodied cognition. Front Psychol. 2011;2. doi:10.3389/fpsyg.2011.00260

~~19~~. 20. Hohol M, Wołoszyn K, Nuerk H-C, Cipora K. A large-scale survey on finger counting routines, their temporal stability and flexibility in educated adults. PeerJ. 2018;6: e5878. doi:10.7717/peerj.5878

~~20.~~ 21. Wasner M, Moeller K, Fischer MH, Nuerk HC. Aspects of situated cognition in embodied numerosity: The case of finger counting. Cogn Process. 2014;15: 317–328. doi:10.1007/s10339-014-0599-z

~~21.~~ 22. Cipora K, Soltanlou M, Reips UD, Nuerk HC. The SNARC and MARC effects measured online: Large-scale assessment methods in flexible cognitive effects. Behav Res Methods. 2019;51: 1676–1692. doi:10.3758/s13428-019-01213-5

~~22~~. 23. Sato M, Lalain M. On the relationship between handedness and hand-digit mapping in finger counting. Cortex. 2008;44: 393–399. doi:10.1016/j.cortex.2007.08.005

~~23~~. 24. Fabbri M, Guarini A. Finger counting habit and spatial-numerical association in children and adults. Conscious Cogn. 2016;40: 45–53. doi:10.1016/j.concog.2015.12.012

~~24.~~ 25. Lindemann O, Alipour A, Fischer MH. Finger Counting Habits in Middle Eastern and Western Individuals: An Online Survey. J Cross Cult Psychol. 2011;42: 566–578. doi:10.1177/0022022111406254

~~25.~~ 26. Lucidi A, Thevenot C. Do not count on me to imagine how I act: behavior contradicts questionnaire responses in the assessment of finger counting habits. Ann

des Telecommun Telecommun. 2014;46: 1079–1087. doi:10.3758/s13428-014-0447-1

~~26.~~ 27. Aleotti S, Di Girolamo F, Massaccesi S, Priftis K. Numbers around Descartes: A preregistered study on the three-dimensional SNARC effect. Cognition. 2020;195: 104111. doi:10.1016/j.cognition.2019.104111

~~27.~~ 28. Winter B, Matlock T, Shaki S, Fischer MH. Mental number space in three dimensions. Neuroscience and Biobehavioral Reviews. Elsevier Ltd; 2015. pp. 209–219. doi:10.1016/j.neubiorev.2015.09.005

~~28.~~ 29. Fischer MH. A hierarchical view of grounded, embodied, and situated numerical cognition. Cogn Process. 2012;13: 161–164. doi:10.1007/s10339-012-0477-5

~~29.~~ 30. Pinto M, Pellegrino M, Lasaponara S, Scozia G, D'Onofrio M, Raffa G, et al. Number space is made by response space: Evidence from left spatial neglect. Neuropsychologia. 2021;154. doi:10.1016/j.neuropsychologia.2021.107773

~~30~~. 31. Shaki S, Fischer MH. Deconstructing spatial-numerical associations. Cognition. 2018;175: 109–113. doi:10.1016/j.cognition.2018.02.022

~~31~~. 32. Gevers W, Lammertyn J, Notebaert W, Verguts T, Fias W. Automatic response activation of implicit spatial information: Evidence from the SNARC effect. Acta Psychol (Amst). 2006;122: 221–233. doi:10.1016/j.actpsy.2005.11.004

~~32.~~ 33. Ito Y, Hatta T. Spatial structure of quantitative representation of numbers: Evidence from the SNARC effect. Mem Cogn. 2004;32: 662–673. doi:10.3758/BF03195857

~~33.~~ 34. Shaki S, Fischer MH. Multiple spatial mappings in numerical cognition. J Exp Psychol Hum Percept Perform. 2012;38: 804–809. doi:10.1037/a0027562

~~34.~~ 35. Fischer MH. Cognitive representation of negative numbers. Psychol Sci. 2003;14: 278–282. doi:10.1111/1467-9280.03435

~~35.~~ 36. Schwarz W, Keus IM. Moving the eyes along the mental number line: Comparing SNARC effects with saccadic and manual responses. Percept Psychophys. 2004;66: 651–664. doi:10.3758/BF03194909

~~36.~~ 37. Hesse PN, Bremmer F. The SNARC effect in two dimensions: Evidence for a frontoparallel mental number plane. Vision Res. 2017;130: 85–96. doi:10.1016/j.visres.2016.10.007

~~37.~~ 38. Liu Di, Cai D, Verguts T, Chen Q. The Time Course of Spatial Attention Shifts in Elementary Arithmetic. Sci Rep. 2017;7: 1–8. doi:10.1038/s41598-017-01037-3

~~38.~~ 39. Myachykov A, Ellis R, Cangelosi A, Fischer MH. Ocular drift along the mental number line. Psychol Res. 2016;80: 379–388. doi:10.1007/s00426-015-0731-4

~~39.~~ 40. Hartmann M, Mast FW, Fischer MH. Spatial biases during mental arithmetic: evidence from eye movements on a blank screen. Front Psychol. 2015;6: 12. doi:10.3389/fpsyg.2015.00012

41. Miklashevsky A, Fischer MH, Lindemann O. Spatial-numerical associations without a motor response? Grip force says ‘Yes.’ Acta Psychol (Amst). 2022;231: 103791. doi:10.1016/j.actpsy.2022.103791

42. Fischer MH, Castel AD, Dodd MD, Pratt J. Perceiving numbers causes spatial shifts of attention. Nat Neurosci. 2003;6: 555–556. doi:10.1038/nn1066

~~41.~~ 43. McCrink K, Dehaene S, Dehaene-Lambertz G. Moving along the number line: Operational momentum in nonsymbolic arithmetic. Percept Psychophys. 2007;69: 1324–1333. doi:10.3758/BF03192949

~~42.~~ 44. Fischer MH, Knops A. Attentional cueing in numerical cognition. Frontiers in Psychology. Frontiers Research Foundation; 2014. doi:10.3389/fpsyg.2014.01381

~~43.~~ 45. Mathieu R, Epinat-Duclos J, Sigovan M, Breton A, Cheylus A, Fayol M, et al. What’s Behind a “+” Sign? Perceiving an Arithmetic Operator Recruits Brain Circuits for Spatial Orienting. Cereb Cortex. 2018;28: 1673–1684. doi:10.1093/cercor/bhx064

~~44.~~ 46. Pinhas M, Shaki S, Fischer MH. Heed the signs: Operation signs have spatial associations. Q J Exp Psychol. 2014;67: 1527–1540. doi:10.1080/17470218.2014.892516

~~45.~~ 47. Masson N, Letesson C, Pesenti M. Time course of overt attentional shifts in mental arithmetic: Evidence from gaze metrics. Q J Exp Psychol. 2018;71: 1009–1019. doi:10.1080/17470218.2017.1318931

48. Pinto M, Pellegrino M, Marson F, Lasaponara S, Doricchi F. Reconstructing the origins of the space-number association: spatial and number-magnitude codes must be used jointly to elicit spatially organised mental number lines. Cognition. 2019;190: 143–156. doi:10.1016/j.cognition.2019.04.032

49. Pinto M, Pellegrino M, Marson F, Lasaponara S, Cestari V, D’Onofrio M, et al. How to trigger and keep stable directional Space–Number Associations (SNAs). Cortex. 2021;134: 253–264. doi:10.1016/j.cortex.2020.10.020

50. Belli F, Felisatti A, Fischer MH. “BreaThink”: breathing affects production and perception of quantities. Exp Brain Res. 2021;239: 2489–2499. doi:10.1007/s00221-021-06147-z

51. Borghi AM, Shaki S, Fischer MH. Abstract concepts: external influences, internal constraints, and methodological issues. Psychol Res. 2022;86: 2370–2388. doi:10.1007/s00426-022-01698-4

52. Matheson HE, Barsalou LW. Embodiment and Grounding in Cognitive. 2018; 1–27. doi:10.1002/9781119170174.epcn310

~~47.~~ 53. Raab M. Judgment, Decision-Making and Embodied Choices. Academic Publisher.; 2021.

~~48.~~ 54. Walsh V. A Theory of Magnitude: The parts that sum to number. The Oxford Handbook of Numerical Cognition. 2015. doi:1093/oxfordhb/9780199642342.013.64

~~49.~~ 55. Walsh V. A theory of magnitude: Common cortical metrics of time, space and quantity. Trends Cogn Sci. 2003;7: 483–488. doi:10.1016/j.tics.2003.09.002

~~50.~~ 56. Loetscher T, Schwarz U, Schubiger M, Brugger P. Head turns bias the brain's internal random generator. Current Biology. Cell Press; 2008. pp. R60–R62. doi:10.1016/j.cub.2007.11.015

~~51.~~ 57. Towse JN, Valentine JD. Random Generation of Numbers: A Search for Underlying Processes. Eur J Cogn Psychol. 1997;9: 381–400. doi:10.1080/713752566

~~52.~~ 58. Shaki S, Fischer MH. Random walks on the mental number line. Exp Brain Res. 2014;232: 43–49. doi:10.1007/s00221-013-3718-7

~~53~~. 59. Winter B, Matlock T. More is up… and right: Random number generation along two axes. Proc Annu Meet Cogn Sci Soc. 2013.

~~54~~. 60. Hartmann M, Grabherr L, Mast FW. Moving along the mental number line: Interactions between whole-body motion and numerical cognition. J Exp Psychol Hum Percept Perform. 2012;38: 1416–1427. doi:10.1037/a0026706

~~55~~. 61. Popper K. The Logic of Scientific Discovery. Basic Books; 1959.

62. Büsch D, Hagemann N, Bender N. Das Lateral Preference Inventory: Itemhomogenität der deutschen Version. Zeitschrift für Sport. 2009;16: 17–28. doi:10.1026/1612-5010.16.1.17

~~56~~. 63. Mayr S, Erdfelder E, Buchner A, Faul F. A short tutorial of GPower. Tutor Quant Methods Psychol. 2007. Available: http://www.psycho.uni-duesseldorf.de/aap/projects/gpower.

~~57.~~ 64. Brysbaert M. How many participants do we have to include in properly powered experiments? A tutorial of power analysis with reference tables. Journal of Cognition. Ubiquity Press; 2019. pp. 1–38. doi:10.5334/joc.72

~~58.~~ 65. Frak V, Nazir T, Goyette M, Cohen H, Jeannerod M. Grip Force Is Part of the Semantic Representation of Manual Action Verbs. Gribble PL, editor. PLoS One. 2010;5: e9728. doi:10.1371/journal.pone.0009728

66. Nazir TA, Hrycyk L, Moreau Q, Frak V, Cheylus A, Ott L, et al. A simple technique to study embodied language processes: the grip force sensor. Behav Res Methods. 2017;49: 61–73. doi:10.3758/s13428-015-0696-7

~~59.~~ 67. Krause F, Lindemann O. Expyriment: A Python library for cognitive and neuroscientific experiments. Behav Res Methods. 2014;46: 416–428. doi:10.3758/s13428-013-0390-6

~~60.~~ 68. Vicario CM. Perceiving numbers affects the internal random movements generator. Sci World J. 2012;2012. doi:10.1100/2012/347068

~~61.~~ 69. Di Bono MG, Zorzi M. The spatial representation of numerical and non-numerical ordered sequences: Insights from a random generation task. Q J Exp Psychol. 2013;66: 2348–2362. doi:10.1080/17470218.2013.779730

~~62~~. 70. Mathôt S, Schreij D, Theeuwes J. OpenSesame: An open-source, graphical experiment builder for the social sciences. Behavior Research Methods. Springer; 2012. pp. 314–324. doi:10.3758/s13428-011-0168-7

~~64.~~ 71. Miklashevsky A. Catch the star! Spatial information activates the manual motor system. Myachykov A, editor. PLoS One. 2022;17: e0262510. doi:10.1371/journal.pone.0262510

~~65.~~ 72. Pulvermüller F, Shtyrov Y, Hauk O. Understanding in an instant: Neurophysiological evidence for mechanistic language circuits in the brain. Brain and Language. 2009. pp. 81–94. doi:10.1016/j.bandl.2008.12.001

~~66~~. 73. Wagenmakers EJ, Marsman M, Jamil T, Ly A, Verhagen J, Love J, et al. Bayesian inference for psychology. Part I: Theoretical advantages and practical ramifications. Psychon Bull Rev. 2018;25: 35–57. doi:10.3758/s13423-017-1343-3

~~67.~~ 74. van Ravenzwaaij D, Wagenmakers EJ. Advantages Masquerading as “Issues” in Bayesian Hypothesis Testing: A Commentary on Tendeiro and Kiers (2019). Psychol Methods. 2022;27: 451–465. doi:10.1037/met0000415

75. Loetscher T, Brugger P. Exploring number space by random digit generation. Exp Brain Res. 2007;180: 655–665. doi:10.1007/s00221-007-0889-0

~~68.~~ 76. JASP Team. JASP (Version 0.16.4)[Computer software]. 2022. Available: https://jasp-stats.org/

~~70.~~ 77. Johari K, Behroozmand R. Premotor neural correlates of predictive motor timing for speech production and hand movement: evidence for a temporal predictive code in the motor system. Exp Brain Res. 2017;235: 1439–1453. doi:10.1007/s00221-017-4900-0

~~71.~~ 78. Engel AK, Maye A, Kurthen M, König P. Where's the action? The pragmatic turn in cognitive science. Trends in Cognitive Sciences. Elsevier Current Trends; 2013. pp. 202–209. doi:10.1016/j.tics.2013.03.006

~~72. Rees M. On the future: Prospects for humanity. Princeton University Press; 2021.~~

~~73.~~ 79. Werner K, Raab M, Fischer MH. Moving arms: the effects of sensorimotor information on the problem-solving process. Think Reason. 2019;25: 171–191. doi:10.1080/13546783.2018.1494630

~~74.~~ 80. Deeney C, O'Sullivan LW. Effects of cognitive loading and force on upper trapezius fatigue. Occup Med (Chic Ill). 2017;67: 678–683. doi:10.1093/occmed/kqx157

~~75. Aravena P, Courson M, Frak V, Cheylus A, Paulignan Y, Deprez V, et al. Action relevance in linguistic context drives word-induced motor activity. Front Hum Neurosci. 2014;8: 163. doi:10.3389/fnhum.2014.00163~~

~~76. Aravena P, Delevoye-Turrell Y, Deprez V, Cheylus A, Paulignan Y, Frak V, et al. Grip Force Reveals the Context Sensitivity of Language-Induced Motor Activity during "Action Words" Processing: Evidence from Sentential Negation. Paterson K, editor. PLoS One. 2012;7: e50287. doi:10.1371/journal.pone.0050287~~

~~77.~~ 81. Rizzolatti G, Riggio L, Sheliga B. Space and Selective Attention. Attention and Performance XV. 1994. doi:10.7551/mitpress/1478.003.0016

82. Almaatouq A, Griffiths TL, Suchow JW, Whiting ME, Evans J, Watts DJ. Beyond Playing 20 Questions with Nature: Integrative Experiment Design in the Social and Behavioral Sciences. Behav Brain Sci. 2022; 1–55. doi:10.1017/S0140525X22002874

~~78.~~ 83. Johansson RS. Sensory Control of Dexterous Manipulation in Humans. Hand and Brain. Elsevier; 1996. pp. 381–414. doi:10.1016/b978-012759440-8/50025-6

~~79.~~ 84. Abolins V, Latash ML. Unintentional force drifts across the human fingers: implications for the neural control of finger tasks. Exp Brain Res. 2022;240: 751–761. doi:10.1007/s00221-021-06287-2

~~80~~. 85. Gibson JJ. The ecological approach to visual perception. Hilisdale, NJ: Lawrence Eribaum Associates; 1986.

~~81.~~ 86. Andres M, Davare M, Pesenti M, Olivier E, Seron X. Number magnitude and grip aperture interaction. Neuroreport. 2004;15: 2773–2777.

~~82.~~ 87. Lindemann O, Abolafia JM, Girardi G, Bekkering H. Getting a Grip on Numbers: Numerical Magnitude Priming in Object Grasping. J Exp Psychol Hum Percept Perform. 2007;33: 1400–1409. doi:10.1037/0096-1523.33.6.1400

~~83.~~ 88. Moretto G, Di Pellegrino G. Grasping numbers. Exp Brain Res. 2008;188: 505–515. doi:10.1007/s00221-008-1386-9

~~84.~~ 89. Hommel B, Müsseler J, Aschersleben G, Prinz W. The Theory of Event Coding (TEC): A framework for perception and action planning. Behav Brain Sci. 2001;24: 849–878. doi:10.1017/S0140525X01000103
